# Immunotherapy Bridge 2018 and Melanoma Bridge 2018: meeting abstracts

**DOI:** 10.1186/s12967-018-1745-7

**Published:** 2019-01-15

## Immunotherapy Bridge 2018

### SITC Session—evolving topics in cancer immunotherapy: tumor microenvironment—oral communications

#### O1 Cancer immunosuppression induced by albumin derived neo-structures

##### Leif Håkansson

###### Canimguide Therapeutics AB, Scheelevägen 2, 223 81 Lund, Sweden

*Journal of Translational Medicine* 2019, **17(Supp 1)**:1

**Background**: Better understanding of immunosuppressor mechanisms is a prerequisite in order to enhance the efficacy of cancer immunotherapy. As the serum concentration of interleukin-6 (IL-6) is increased in the majority of advanced cancer patients, the occurrence of unknown factors inducing this cytokine was further explored.

**Materials and methods**: Immunoregulatory albumin neo-structures were identified by adsorption to immune cell receptors, further analysed by 2-D gel electrophoresis, MALDI-TOF and sequencing according to Edman. Binding of this neo-structure to LFA-1 was investigated using immunohistochemistry. Production of IL-6 was determined by ELISA.

**Results**: Proteolytic fragmentation or denaturation of normal serum albumin was found to generate conformational changes, neo-structures, with the capacity to induce IL-6 production by normal mononuclear blood cells (PBMC). Analysis of albumin sequences identified a sequence inducing IL-6. Further analysis of immune cell binding albumin neo-structures also identified a potent immunosuppressor, P3028, binding to LFA-1 and CD25 on immune cells and thereby inhibiting lymphocyte proliferation and migration and NK-cell cytotoxicity. The immunosuppressor 3028 is a physiological blocker of LFA-1, a β2-integrin of fundamental importance for multiple activities of the immune system, crucial for immune mediated cancer control. Blockade of LFA-1 inhibits: Initiation of an immune response, lymphocyte proliferation, lymphocyte recruitment to tumours, migration of these cells within tumours and their cytolytic activity. Blocking of 3028 either by antibodies directed to this structure or by a complementary binding peptide (P28R) reverse cancer related immunosuppression in an in vitro lymphocyte proliferation assay. 3028-structures are frequently found to block LFA-1 in animal as well as human tumours and ex vivo treatment of tumour sections with P28R efficiently unblock this receptor. The 3028-structure is expressed in all types of tumours studied so far, e.g. breast, colon, prostate cancer, squamous cell cancer of the oral cavity, renal cell carcinoma and melanoma. Treatment of spontaneous tumours in dogs by injecting P28R subcutaneously, blocking the immunosuppressor 3028, results in a significantly enhanced inflammatory infiltrate, eradication of tumour cells and an almost complete histopathological regression after a single injection within 5 days.

**Conclusions**: This investigation identified and characterized a physiological inhibitor of LFA-1, of fundamental importance for multiple activities of the immune system, crucial for immune mediated cancer control. Blocking of the immunosuppressor 3028 is a new therapeutic strategy for reversal of cancer related immunosuppression.

#### O2 Targeted next generation sequencing for the evaluation of tumor mutation burden

##### Francesca Fenizia^1^, Raffaella Pasquale^1^, Cristin Roma^1^, Francesca Bergantino^1^, Nicoletta Chicchinelli^1^, Paolo Graziano^2^, Gerardo Botti^3^, Fabiana Tatangelo^3^, Giosuè Scognamiglio^3^, Matilde Lambiase^1^, Alessia Iannaccone^1^, Nicola Normanno^1^

###### ^1^Cell Biology and Biotherapy Unit, Istituto Nazionale Tumori “Fondazione G. Pascale”-IRCCS, Naples, Italy; ^2^Unit of Pathology, IRCCS “ Casa Sollievo della Sofferenza” Hospital, San Giovanni Rotondo, Foggia, Italy; ^3^Pathology Unit, Istituto Nazionale Tumori “Fondazione G. Pascale”-IRCCS, Naples, Italy

*Journal of Translational Medicine* 2019, **17(Supp 1)**:2

**Background:** Tumour mutation burden (TMB) is defined as the total number of mutations per coding area of a tumour genome and it has been associated with clinical response to Immune checkpoint inhibitors (IOs) in different tumor types, including colorectal cancer (CRC) with mismatch repair deficiency [1]. Targeted next generation sequencing (TS) can be an alternative to whole exome sequencing (WES) to determine TMB in clinical practice [2]. We performed TMB analysis on tumor cell lines and CRC samples and compared the results with microsatellite instability (MSI) status to evaluate the clinical robustness of a TS approach.

**Materials and methods:** Genomic DNA (gDNA) was extracted from CRC cell lines using the QIAgen DNeasy Blood&Tissue kit. To obtain gDNA from FFPE CRC samples, we optimized an extraction method that overcomes fixation issues, which cause an increase of deamination mutations, thus altering TMB values. The extraction workflow includes enzymatic removal of deamination artifacts, which result in sequencing errors. For both samples, TMB libraries were prepared with the Oncomine Tumor Mutation Load Assay (Thermofisher) and sequenced on the Ion S5 platform. TMB was defined as the total number of somatic SNVs divided by number of bases with sufficient coverage. MSI status was evaluated by means of the Bethesda panel and the Idylla MSI assay (Biocartis) [3].

**Results**: We first evaluated the TMB in 8 cell lines and the results were correlated with the massively parallel sequencing data from > 1600 genes performed on the same cell lines available on cBioPortal [4], demonstrating the ability of the panel to infer WES-TMB (R^2^ = 0.979). In our cell lines, a strong correlation between TMB and MSI status was also observed. The optimized extraction workflow allowed to overcome DNA damage due to fixation and obtain successful sequencing in all the FFPE CRC samples analyzed. TMB evaluation performed on 32 CRC tumor tissues from the Pascale Institute biobank was compared to the MSI results. Significant differences were found in TMB values of MSI-High (MSI-H, n = 9, median value: 40.25) versus microsatellite stable (MSS) cases, as expected (n = 23, median value: 10.97) (Fig. 1; Mann–Whitney test: P < 0.0001).

**Fig. 1:**
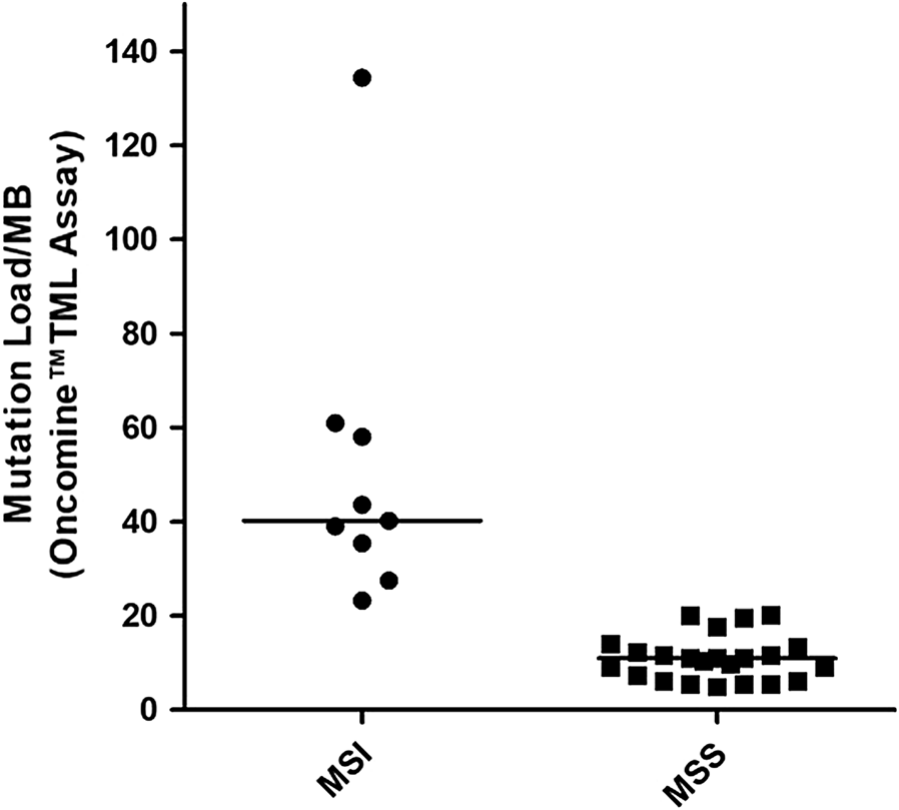
TMB distribution according to MSI status

**Conclusions:** These data suggest that the developed workflow for TMB testing provides results in line with the expected tumor mutational load. Targeted sequencing can represent a valid approach that might be translated into clinical practice to ensure that patients receive an appropriate TMB test.

**References**

1. Yarchoan M, Hopkins A, Jaffee EM. Tumor Mutational Burden and Response Rate to PD-1 Inhibition. N Engl J Med. 2017;377(25):2500–1.
2. Chalmers ZR, Connelly CF, Fabrizio D, Gay L, Ali SM, Ennis R, et al. Analysis of 100,000 human cancer genomes reveals the landscape of tumor mutational burden. Genome Med. 2017;9(1):34.
3. Umar A, Boland CR, Terdiman JP, Syngal S, de la Chapelle A, Ruschoff J, et al. Revised Bethesda Guidelines for hereditary nonpolyposis colorectal cancer (Lynch syndrome) and microsatellite instability. J Natl Cancer Inst. 2004;96(4):261–8.
4. Barretina J, Caponigro G, Stransky N, Venkatesan K, Margolin AA, Kim S, et al. The Cancer Cell Line Encyclopedia enables predictive modelling of anticancer drug sensitivity. Nature. 2012;483(7391):603–7.

#### O3 Activation of immune response in refractory patients to standard treatment

##### Marcella Occelli^1^, Andrea Abbona^2^, Dario Sangiolo^3,4^, Cristiana Lo Nigro^1^, Ornella Garrone^1^, Antonella Falletta^2^, Merlotti Anna^1^, Chiara Varamo^3,4^, Massimo Aglietta^3,4^, Loretta Gammaitoni^4^, Martino Monteverde^2^, Marco Merlano^1,2^

###### ^1^Ospedale Santa Croce e Carle Cuneo, Cuneo, Italy; ^2^Laboratorio di Oncologia Traslazionale, Fondazione Arco Cuneo, Cuneo, Italy; ^3^Department of Oncology, University of Torino, Torino, Italy; ^4^Candiolo Cancer Institute FPO-IRCCS, Torino, Italy

*Journal of Translational Medicine* 2019, **17(Supp 1)**:3

**Background:** The TRANSLATE project started in 2016 to test the immune effects of metronomic cyclophosphamide, daily low-dose IL-2 every other week, and a single flash of radiotherapy (RT) in peripheral blood.

The rationale is based on self vaccination induced by radiotherapy (RT) 8 Gy single fraction on one metastatic lesion, T cells expansion by IL-2 treatment and selective Tregs down regulation by lowdose cyclophosphamide administration.

**Materials/methods:** We enrolled patients with end-stage breast, colon, kidney and prostate cancer. Analysis was performed at baseline, the day after RT, after 28 days from treatment start and at disease progression. Assay focused on Tregs, CD8+, NK, MDSC, CD3-PD1, IL-2, IL-4, IL-5, IL-6, IL-10, IL-12, IL-13, IL-17a, TNFα, IFNγ, TGFβ. We divided patients into two groups depending on the time of disease progression (A > 3 months; B, < 3 months). We report preliminary data with the aim to show the changes observed post-RT in 20 pts.

**Results:** At baseline, group B had higher rates of CD3-PD1, higher IL-2, IL-13, TNFα, and TGFβ; group A had higher IFN γ, IL-4, and IL-12. After RT, we observed a difference between the two groups in the rates of CD3-PD1 (lower in group B) and Tregs (higher in group A). Among cytokines, only TNFα reached a statistical significance (higher in group B). We also observed that TGFβ and IL-6 were higher in group B and IFNγ was higher in group A. The longitudinal analyses showed that CD3-PD1 remained stable between basal and post-RT in group A but decreased in group B. Tregs marginally increased in group A. TNFα, TGFβ, IL-4, IL-6, and IL-12 increased in group B. IL-4 decreased in group A and IL-6 and IL-12 also marginally decreased in the same group. IFNγ slightly increases in group A.

**Conclusions:** The limited number of patients reduced the interpretation of the study. However, following RT a positive trend of Th2 cytokines is observed in patients with early progressing disease, without the expected surge of IFNγ that was instead observed in patients with better outcome. Additional analyses are in progress and will be presented.

#### O4 Dysregulation of immune modulating molecules and signaling pathways in dendritic cells/DC-based vaccines generated from advanced-stage melanoma patients

##### Deena M Maurer^1^, Patricia Santos^2^, John M Kirkwood^2^, Ahmad A. Tarhini^7^, Hussein A. Tawbi^5^, David Stroncek^6^, Ping Jin^6^, and Lisa H Butterfield^1,2,3,4^

###### ^1^Department of Immunology, University of Pittsburgh, Pittsburgh PA; ^2^Department of Medicine, University of Pittsburgh Cancer Institute, Pittsburgh PA; ^3^Department of Surgery, University of Pittsburgh Cancer Institute, Pittsburgh PA; ^4^Department of Clinical and Translational Science, University of Pittsburgh, Pittsburgh PA; ^5^MD Anderson Cancer Center, University of Texas, Houston TX; ^6^Department of Transfusion Medicine, National Institutes of Health, Bethesda MD; ^7^Lerner School of Medicine, Case Western Reserve University, Cleveland OH

*Journal of Translational Medicine* 2019, **17(Supp 1)**:4

**Background:** Stage IV melanoma has a 5-year survival rate of only 15–20%. Immune checkpoint blockade has shown therapeutic efficacy in a subset of melanoma patients, often those with pre-existing antitumor immunity. Therapeutic vaccines targeting melanoma-associated antigens are commonly immunogenic, but only rarely effective in promoting clinical responses, suggesting a clear need for further improvement.

**Materials and methods:** To promote strong anti-tumor immune responses in melanoma patients, we created a vaccine consisting of autologous dendritic cells (DC) transduced ex vivo with a recombinant adenovirus encoding three shared melanoma antigens: Tyrosinase, MART-1, and MAGE-A6 (TMM2). Monocyte-derived DC were first matured with IFN-γ + LPS, and then transduced with recombinant adenovirus encoding TMM2 and administered to patients (n = 35) 3× via bi-weekly intradermal injections in a Phase I trial. Human microarrays were used to analyze gene expression profiles of patient immature, mature, and adenovirus-transduced DC.

**Results:** Genomic analyses revealed that melanoma patient (but not healthy donor [1]) DC exhibit a significant increase in expression of transcripts encoding immunosuppressive molecules, such as IDO1 and TXN after ex vivo maturation and viral transduction, when compared to individual-matched immature DC. DC-associated transcripts correlating significantly with clinical outcome include LAMP1, DDO, CLEC4A and NLRP2 linked to antigen-presentation, aspartate degradation, plasmacytoid DC and an inhibitor of TBK1/Type-1 IFN signaling, respectively.

Protein-based analyses indicate ICOSL downregulation on the surface of immature, mature, and transduced DC in melanoma patients versus HD. NFkB signaling, known to regulate ICOSL expression in DC, appears selectively dysregulated in mDC generated from melanoma patients versus HD.

**Conclusions:** Our profiling data suggest deficiencies in NFkB and Type-1 IFN signaling and costimulatory molecule expression (ICOS-L), as well as elevated expression of immunosuppressive gene products (IDO1, TXN), may serve to limit the immunostimulatory capacity of melanoma patient-derived DC and derivative DC-based vaccines. These data provide clues for targeted manipulation of patient DC to develop improved vaccines implementing DC for the treatment of advanced-stage melanoma patients.

Trial Registration Identifier NCT01622933.

**Reference**

1. Jin, P., T. H. Han, J. Ren, S. Saunders, E. Wang, F. M. Marincola, and D. F. Stroncek. 2010. Molecular signatures of maturing dendritic cells: implications for testing the quality of dendritic cell therapies. J. Transl. Med. 8: 4.

#### O5 Class I HDAC inhibitor domatinostat beneficially affects phenotype and functionality of T cells in the tumor microenvironment, and synergizes with PD-1/LAG3 checkpoint blockade

##### Svetlana Hamm, Ulrike Parnitzke, René Bartz, Frank Hermann

###### SC AG, Planegg-Martinsried, Germany

*Journal of Translational Medicine* 2019, **17(Supp 1)**:5

**Background:** Anti-PD-1 plus anti-LAG3 therapy was well tolerated in melanoma patients and has shown clinical activity in patients progressive on PD-1 therapy. However, a significant proportion of patients still exhibit a priori resistance or suffer from disease progression. Domatinostat, a selective HDAC class I inhibitor, has been preclinically reported to upregulate MHC molecules, tumor-associated antigen expression, and increase inflammatory signature and T cell infiltration into tumors. Here, we provide preclinical data on the triple combination of domatinostat, anti-PD-1 plus anti-LAG3 antibodies.

**Materials and methods:** Anti-tumoral efficacy and the impact on tumor microenvironment were analyzed in a syngeneic C38 model with a low response rate to anti-PD-1 and no response to anti-LAG3 treatment, reflecting refractory clinical situation. Clinical equivalent dose of domatinostat was used to ensure translational relevance.

**Results:** Domatinostat and anti-PD1 in monotherapy reduced C38 tumor growth resulting in 10% and 25% regressions, respectively. The combination of domatinostat plus anti-PD-1 antibody resulted in a stronger tumor growth control with 60% regressing tumors. Anti-LAG3 treatment alone or in combination with either domatinostat or anti-PD-1 did not show added anti-tumoral efficacy. However, addition of domatinostat to anti-PD-1+ anti-LAG3 double combination resulted in a higher anti-tumor activity with a tumor regression rate of 80%. FACS analysis revealed that domatinostat increased expression of MHC class I molecules on tumor cells, and MHC class II molecules on tumor cells, Ly6G+ myeloid derived suppressor cells and M1 macrophages. Domatinostat-mediated upregulation of MHC class II and co-stimulatory molecules [Bretz et al. AACR 2017] allows to hypothesize that the addition of an anti-LAG3 antibody, which blocks detrimental effect of MHC class II/LAG3 engagement on T cell activity, may beneficially affect T cell response by supporting T helper cell activation and function. Consistently, in combination with both checkpoint inhibitors domatinostat strongly enhanced the proportion of proliferating cytotoxic T cells (CTLs) in the tumors and reduced exhaustion phenotype of CTLs and T helper cells.

**Conclusions**: Domatinostat has previously demonstrated beneficial immunomodulatory effects in syngeneic preclinical tumor models in combination with immunostimulating agents as well as with immune checkpoint blockade. Currently, domatinostat is in clinical evaluation in combination with pembrolizumab in advanced melanoma patients refractory or non-responding to anti-PD-1 (“SENSITIZE” study; NCT03278665). The triple combination of domatinostat, anti-PD-1 and anti-LAG3 in the C38 syngeneic mouse model resulted in a high rate of complete responses, superior to any double combination, suggesting a favorable synergy justifying and warranting clinical investigation.

## Immunotherapy Bridge 2018

### Trends in immunotherapy session—oral communications

#### O6 Synergistic potentiation of the anti-metastatic effect of anti EGFR mAb by its combination with immunotherapies targeting the ganglioside NGcGM3

##### A. González^1^, A. López^1^, V. Segatori^1^, M. C. Barroso^1^, R. Blanco^1^, M. R. Gabri^2^, K. León^1^, A. Carr^1^

###### ^1^Center of Molecular Immunology (CIM), Atabey, Playa, Havana, Cuba; ^2^Laboratory of Molecular Oncology, Quilmes National University, Buenos Aires, Argentina

*Journal of Translational Medicine* 2019, **17(Supp 1)**:6

**Background:** Epidermal growth factor receptor (EGFR) plays an important role in cancer progression. However, the impact of anti-EGFR therapies on patients overall survival has been limited mainly by the emergence of different forms of tumor resistance[1]. N-glycolyl variant of GM3 ganglioside (NGcGM3) is specific antigen expressed in some tumors, it has received some attention as a privileged target for cancer therapy [2]. This ganglioside has been associated with a poor prognostic in colon and lung cancer [3,4]. Several reports have documented a functional relationship between GM3 and EGFR at tumor cell membrane. GM3 physically associates to EGFR inhibiting its ligand depend phosphorylation, but it also facilitates an alternative/compensatory signaling cascade mediated by Uroquinase Plasminogen Activator Receptor (uPAR) and integrin α5β1 interaction with membrane reduction of NAcGM3 ganglioside [5]. However, no definite information happens on the difference between N-glycolyl and N-Acetyl variants of GM3 regarding this interaction [6].

**Materials and methods:** The impact study of a combination of immunotherapies against these two “physically and/or functional related targets NGcGM3 and EGFR” were performed in two murine lung metastasis models: (Lewis lung carcinoma (3LL-D122) in C57BL/6 and mammary carcinoma (4T1) in BALB/c). 7A7 murine monoclonal antibody (mMAb) against murine EGFR was the therapy selected to this molecule [7]. While, two therapies against NGcGM3 ganglioside were evaluated NGcGM3/VSSP vaccine (NeuGcGM3 was incorporated in the outer membrane protein complex of *Neisseria meningitidis* bacteria to form very small size proteoliposomes (VSSP)) [8] or 14F7 mMAb is an IgG1 vs NGcGM3 [9].

**Results:** A clear synergistic effect on survival of the combination immunotherapies against both targets in either tumor models was found. Additionally, the assessment of treated metastasis demonstrated that NGcGM3/VSSP vaccine reduce Src/FAK/Stap3 cascade but combination therapy turns off more the signaling through both EGFR and uPAR/α5β1 integrin pathway, reduction of NGcGM3 ganglioside membrane expression and angiogenesis process, in parallel infiltrated of NK1.1 cellular was observed. Nevertheless, survival effect was dependent of T CD8+, TCD4+ and NK1.1 cells.

**Conclusion.** Overall, our results support the potential combination of anti EGFR antibodies with therapies targeting NGcGM3 to increase their efficacy in future clinical trials.

**References**

1. Brand TM, Lida M, Wheeler DL. Molecular mechanisms of resistance to the EGFR monoclonal antibody cetuximab. Cancer Biol Ther. 2011; 11:777–92.
2. Fernandez LE, Gabri MR, Guthmann MD, Gomez RE, Gold S, Fainboim L, Gomez DE, Alonso DF. NGcGM3 ganglioside: a privileged target for cancer vaccines. Clin Dev Immunol. 2010; 2010:814397.
3. Lahera T, Calvo A, Torres G, Rengifo CE, Quintero S, Arango MC, Danta D, Vázquez JM, Escobar X, Carr A. Prognostic role of 14F7 mab immunoreactivity against N-Glycolyl GM3 ganglioside in colon cancer. J Oncol. 2014; 2014:482301.
4. Blanco R, Domínguez E, Morales O, Blanco D, Martínez D, Rengifo CE, Viada C, Cedeño M, Rengifo E, Carr A. Prognostic significance of N-Glycolyl GM3 ganglioside expression in non-small cell lung carcinoma patients: new evidences. Pathol Res Int. 2015; 2015:132326.
5. Wang XQ, Sun P, Go L, Koti V, Fliman M, Paller AS. Ganglioside GM3 promotes carcinoma cell proliferation via urokinase plasminogen activator-induced extracellular signal-regulated kinase-independent p70S6 kinase signaling. J Invest Dermatol. 2006; 126:2687–96.
6. Hayashi N, Chiba H, Kuronuma K, Go S, Hasegawa Y, Takahashi M, Gasa S, Watanabe A, Hasegawa T, Kuroki Y, Inokuchi J, Takahashi H. Detection of N-glycolyated gangliosides in non-small-cell lung cancer using GMR8 monoclonal antibody. Cancer Sci. 2013; 104:43–47.
7. Garrido G, Sanchez B, Rodriguez HM, Lorenzano P, Alonso D, Fernandez LE. 7A7 MAb: a new tool for the pre-clinical evaluation of EGFR-based therapies. Hybrid Hybridomics. 2004; 23:168–75.
8. Estevez F, Carr A, Solorzano L, Valiente O, Mesa C, Barroso O, Sierra GV, Fernandez LE. Enhancement of the immune response to poorly immunogenic gangliosides after incorporation into very small size proteoliposomes (VSSP). Vaccine. 2000; 18:190–197.
9. Carr A, Mullet A, Mazorra Z, Vázquez AM, Alfonso M, Mesa C, Rengifo E, Pérez R, and Fernández LE. A Mouse IgG1 Monoclonal Antibody Specific for N-Glycolyl GM3 Ganglioside Recognized Breast and Melanoma Tumors. Hybridoma 2000; 19: 241–247.

#### O7 Emotional disturbances, social outcome and neurocognitive function in advanced melanoma survivors treated with pembrolizumab

##### Anne Rogiers^1,2,3^, Jennifer De Cremer^3^, Gil Awada^2,3^, Julia Katharina Schwarze^2,3^, Laila Ben Salama^2,3^, Peter Theuns^3^, Mark De Ridder^2,3^, Bart Neyns^2,3^

###### ^1^Centre Hospitalier Universitaire Brugmann, ^2^Universitair Ziekenhuis Brussel, ^3^ Vrije Universiteit Brussel

*Journal of Translational Medicine* 2019, **17(Supp 1)**:7

**Introduction:** An increasing proportion of advanced melanoma patients (pts), treated with immunotherapy and/or BRAF/MEK-inhibitors, achieve long-term survival ^1 2^. Long-term psychosocial outcome and neurocognitive function have not been studied yet. The objective of this study is to investigate the psychological burden related to the traumatic experiences of the disease, the psychosocial outcome, Health Related Quality of Life (HRQoL) and neurocognitive function in survivors treated with pembrolizumab (PEMBRO).

**Materials and methods:** Pts with advanced melanoma (AJCC stages IIIC or IV) who were in remission for at least 1 year after treatment initiation with PEMBRO, were eligible for this ongoing single-center trial. Data on HRQoL, psychosocial outcome and neurocognitive function (NCF) were collected using 5 validated questionnaires, a semi-structured psychiatric examination (SSPE) and computer-based NCF testing.

**Results:** Test results from 26 pts (8 M/18 F; median age 55 years [range 28–86]) were analyzed. Median time since diagnosis of stage IIIC-IV melanoma was 34 months (range 12–84). Mean EORTC-QLQ-C30^3^ Global Score was significant lower than the European Mean of healthy pts^4^ (t (25) = 2.810, p = 0.009). The psychiatric examination revealed that all survivors reported fear of recurrence, of whom 14 (54%) worried daily about their disease. Irritability with impact on social functioning was a prominent complaint in 11 pts (42%). Thirteen pts (50%) received a message of no hope at diagnosis of metastatic disease which had a persistent psychological impact. Eight pts (31%) had elevated scores on the Hospital Anxiety Depression Scale: 6 pts (23%) had severe anxiety with comorbid depression, 1 pt moderate depression, 1 pt moderate anxiety. Three pts (11%) evoked active suicidal ideation; one of them made a suicide attempt; 4 pts (15%) expressed a strong wish to die of whom 2 pts made a request for euthanasia during the disease process. Eight pts (31%) reported worrying about their family; 5 pts (19%) relational problems and 10 pts (38%) financial problems related to the disease. Thirteen pts (50%) had elevated scores on the Cognitive Failure Questionnaire; 12 pts were still on PEMBRO. Eleven pts (42%) had elevated scores on the Fatigue Severity Scale, 9 pts were still on treatment.

**Conclusions:** These results indicate that advanced melanoma survivors treated with PEMBRO are at high risk for suffering from severe emotional disturbances and neurocognitive symptoms with impact on their social functioning and subjective wellbeing. Timely detection of psychosocial and neurocognitive problems in order to offer adapted care are indicated.

**References**

1. Schachter, J., A. Ribas, et al. (2017). Pembrolizumab versus ipilimumab for advanced melanoma: final overall survival results of a multicentre, randomised, open-label phase 3 study (KEYNOTE-006). Lancet.
2. Wolchok, J. D., V. Chiarion-Sileni, et al. (2017). Overall Survival with Combined Nivolumab and Ipilimumab in Advanced Melanoma. N Engl J Med.
3. Aaronson NK, Ahmedzai S, et al. (1993). The European Organisation for research and treatment of cancer QLQ-C-30:A quality of life instrument for use in international clinical trials in oncology. Journal of National Cancer Institute.
4. Hinz A, Singer S., Brâhler E. (2014) European reference values for the quality of life questionnaire EORTC QLQ-C30: Results of a German investigation and a summarizing analysis of six European general population normative studies. Acta Oncologica.

#### O8 Beyond corticotherapy: how to manage severe immune adverse events that do not respond to corticotherapy?

##### Teresa Amaral^1,2^; Carola Berking^3^, Carmen Loquai^4^, Lucie Heinzerling^5^, Lisa Zimmer^6^, Selma Ugurel^7^, Claus Garbe^1^, Thomas Eigentler^1^

###### ^1^Center for Dermatooncology, Department of Dermatology, University Hospital Tuebingen, Germany; ^2^Portuguese Air Force Health Direction, Lisbon, Portugal; ^3^Department of Dermatology and Allergy, University Hospital Munich, Ludwig-Maximilian University, Munich, Germany; ^4^Department of Dermatology, University Medical Center Mainz, Germany; ^5^Department of Dermatology, University Hospital of Erlangen, Germany; ^6^Department of Dermatology and Venereology, Skin Tumor Centre, Study Outpatient Unit, University Hospital Essen, Germany; ^7^Department of Dermatology, Venereology and Allergology, University Hospital Essen, Germany

*Journal of Translational Medicine* 2019, **17(Supp 1)**:8

**Background:** The treatment of patients with metastatic melanoma has changed considerably, particularly with the approval of targeted and immunotherapies, which have improved patients’ outcomes. Managing patients receiving these therapies is challenging, since the safety profile is significantly different from the therapies previously used. In order to address the treatment of these different adverse events, new guidelines were published [1]. In most cases corticoids are recommend as first-line approach. However, in a small group of patients experiencing severe toxicities treatment with corticosteroids is not sufficient.

**Patients and methods:** We conducted a retrospective multicenter (6 centers) survey including patients who experienced immune-related adverse events (irAE) and didn´t respond to corticotherapy. Gender, age, type of immunotherapy, type of adverse event, time of onset, duration, supportive therapy received and outcome were documented (Ethical commission approval—699/2017BO2).

**Results:** Fourteen males and ten females were included. The median age at time of advanced disease was 59 years old (min: 31; max: 81). Eighteen patients were diagnosed with cutaneous melanoma, four with uveal melanoma and two with mucosal melanoma. Twelve patients received ipilimumab, eight received combined immunotherapy (CTLA-4 and PD-1 antibodies) and four were treated with PD-1 inhibitor. The most common irAE was colitis (twelve cases), followed by hepatitis (seven cases) and arthritis (two cases). Other reported irAEs included one case of thyroiditis, pyoderma gangraenosum, myositis and Guillain–Barre syndrome. Twenty-five irAE were classified as Grade 3 (CTCAE v.4) and the majority (thirteen cases) occurred after two cycles. The median time to irAE onset was 7 weeks (min: 1; max: 21), and all patients received corticotherapy as first supportive therapy. The median duration of corticotherapy was 12 weeks (min: 1; max: 35).

Infliximab was the most used therapy (fifteen cases) followed by mycophenolate mofetil (six cases). Methotrexate and IVIG were used in two cases each. Another subsequent therapy was required in three cases and four immunosuppressive treatments in one case. In nineteen cases a complete recovery of the irAEs was observed. The median time to resolution was 11 weeks (min: 1; max: 40).

**Conclusions:** For a minority of patients, solely therapy with corticosteroids is not sufficient. The interdisciplinary exchange about these toxicities is crucial, since experiences of individual therapy courses can be adapted to other patients.

**Reference**

1. Haanen, J.B.A.G., et al., *Management of toxicities from immunotherapy: ESMO Clinical Practice Guidelines for diagnosis, treatment and follow*-*up†.* Annals of Oncology, 2017. **28**(suppl_4): p. iv119–iv142.

#### O9 Evaluation of efficacy and safety of retreatment or concurrent immunoradiotherapy after progression in metastatic melanoma patients previously treated with nivolumab

##### Yumi Nonomura^1,2^, Motoo Nomura^3^, Atsushi Otsuka^2,4^, Michio Yoshimura^5^, Tomohiro Kondo^3^, Hiroki Nagai^3^, Yo Kaku^2^, Shigemi Matsumoto^3^, Manabu Muto^3^, Mitchell Levesque^1^, Reinhard Dummer^1^

###### Department of Dermatology, University Hospital Zürich Skin Cancer Center, Zürich, Switzerland; ^2^Department of Dermatology, Kyoto University Graduate School of Medicine, Kyoto, Japan; ^3^Department of Therapeutic Oncology, Kyoto University Graduate School of Medicine, Kyoto, Japan; ^4^Translational Research Department for Skin and Brain Diseases, Kyoto University Graduate School of Medicine, Kyoto, Japan; ^5^Department of Radiation Oncology and Image‑applied Therapy, Kyoto University Graduate School of Medicine, Kyoto, Japan

*Journal of Translational Medicine* 2019, **17(Supp 1)**:9

**Background:** Immunotherapy and targeted therapies have improved the prognosis of patients with metastatic melanoma [1, 2, 3]. The objective of this study was to evaluate (1) the efficacy and safety of retreatment with nivolumab and (2) that of concurrent immune checkpoint inhibitor therapy and radiotherapy (immunoradiotherapy) in patients with metastatic melanoma after progression on nivolumab.

**Patients and methods:** (1) A retrospective review was performed on eight consecutive metastatic melanoma patients retreated with nivolumab who progressed on previous nivolumab. These patients received nivolumab 2 mg/kg every 3 weeks. Best responses to each treatment were assessed using RECIST 1.1. (2) A retrospective review was performed on 16 consecutive patients with metastatic melanoma treated with concurrent immunoradiotherapy after progression on nivolumab. Best responses to immunoradiotherapy were assessed either inside or outside of the radiation fields. The target lesions ratio (the sum of the diameters of the target lesions inside the irradiated fields/all target lesions) was also assessed.

**Results:** (1) Three out of eight patients received chemotherapy before first nivolumab. The median first nivolumab treatment period was 4.1 months. Three (37.5%) patients achieved a partial response and three (37.5%) patients achieved stable disease as their best response. Between first and second nivolumab, patients were treated with ipilimumab (n = 6), vemurafenib (n = 1), or no other medical treatment (n = 1). Four patients received radiation therapy. The median second nivolumab treatment period was 4.3 months. Two (25%) patients who received second nivolimab achieved a partial response and three (37.5%) patients achieved stable disease as their best response. Among the four patients treated with ipilimumab and radiotherapy between first and second nivolumab, the response rate was 50% and the disease control rate was 75%. (2) Among the patients, seven received ipilimumab and radiotherapy (Ipi-RT), six received nivolumab and radiotherapy (Nivo-RT), and three sequentially received Ipi-RT and Nivo-RT. As shown in Table 1, the overall response rate (all patients regardless of inside or outside radiation fields) was 30%. The response rate inside the radiation fields was 68.8% for all patients combined. The response rates of Ipi-RT and Nivo-RT inside the radiation fields were 37.5 and 100% (P = 0.03), respectively. Grade 3 adverse events were observed in three patients treated with Ipi-RT. The target lesions ratio was a predictive marker of disease control rate among patients treated with Nivo-RT.

**Table 1:** Efficacy of concurrent immunoradiotherapy

	All			Nivo-RT			Ipi-RT		
	All	Inside	Outside	All	Inside	Outside	All	Inside	Outside
CR	1	1	1	0	0	0	1	1	1
PR	5	10	0	5	8	0	0	2	0
SD	2	4	7	1	0	6	1	4	1
PD	12	1	12	4	0	4	8	1	8
NE	0	4	0	0	2	0	0	2	0
Response rate (%)	30.0	68.8	5.0	50.0	100.0	0.0	10.0	37.5	10.0
Disease control rate (%)	40.0	93.8	40.0	60.0	100.0	60.0	20.0	87.5	20.0

**Conclusions:** This study showed that retreatment with nivolumab or concurrent immunoradiotherapy is an option for patients with metastatic melanoma after progression on nivolumab.

**Consent to publish:** Informed consent including consent to publish was obtained from all individual participants included in the study.

**References**

1. Hodi FS, O’Day SJ, McDermott DF et al. Improved survival with ipilimumab in patients with metastatic melanoma. N Engl J Med. 2010; 363:711–723.
2. Weber JS, D’Angelo SP, Minor D et al. Nivolumab versus chemotherapy in patients with advanced melanoma who progressed after anti-CTLA-4 treatment (CheckMate 037): a randomised, controlled, open-label, phase 3 trial. Lancet Oncol. 2015; 16:375–384.
3. Robert C, Long GV, Brady B et al. Nivolumab in previously untreated melanoma without BRAF mutation. N Engl J Med. 2015; 372:320–330.

#### O10 Nivolumab flat dose changes clinical practice and therapy costs

##### Teresa Tramontano, Maria R. Sarno, Imma De Stasio, Ida Palazzo, Piera Maiolino

###### S.C. Farmacia, Istituto Nazionale Tumori - IRCCS Fondazione G. Pascale, Napoli, Italia

*Journal of Translational Medicine* 2019, **17(Supp 1)**:10

**Background:** A new dosing schedule, flat dose, was recently approved for Nivolumab. The recommended flat dose is 240 mg every 2 weeks or 480 mg every 4 weeks depending on the indication, instead of the oldest one, 3 mg/kg every 2 weeks. Since the introduction of Nivolumab^®^ in therapy the Centralized Unit for Handling Antineoplastic of National Cancer Institute “G. Pascale” of Naples planned a drug day to optimize the use in reducing waste and costs. The aim of this work was to analyse drug consumption and cost of Nivolumab^®^ administration before and after flat dose regimen introduction in clinical practice.

**Materials and methods:** Nivolumab flat dose was introduced in therapy on 02/05/2018, and we performed an hypothetical comparison, in consumptions and costs, between the flat dose and no flat dose regimen. This evaluation was performed taking in account patients treated in June 2018 in flat dose regimen. All data, number of patients treated, consumption and cost were collected by data base of Clinical Pharmacy of the hospital. In addition the effectiveness of drug day was evaluated by comparing the number of drug vials really used and those that should be used without the drug day.

**Results**: In June 2018, 110 patients were treated with nivolumab for authorized indications and 1 patient was treated with nivolumab as off-label treatment. For this patient number the total drug consumption was 48240 mg in flat dose regimen with a cost of €570545. In no flat dose regimen, for the same patients the consumption should have been 35279 mg with a cost of €417252. Therefore were used 12961 mg (approximately 130 vials of 100 mg) more than the oldest per kilo dose and an extra expenditure of €153,292.

Comparing the dispensed drug in drug day to the drug really bought we saved 4993 mg (approximately 50 vials of 100 mg) with an economy of €59,058; this is related to the overfill of injecting drug vials, corresponding to about 10–12 mg over the declared amount of drug, as prescribed by F.U. XII Edition (2.9.17) and by FDA guidelines on the filling volume in excess of vials.

**Conclusions:** Nivolumab flat dose recently introduced changed not only clinical practice but also therapy costs in particular the doses were increased so the costs were higher. However drug day allows to recover all drug residues, so it is a very effective tool for the containment of pharmaceutical costs.

## Immunotherapy Bridge 2018

### Drivers of immune responses session—oral communications

#### O11 Prognostic differences between male and female patients in virus-negative Merkel cell carcinoma

##### Hannah Björn-Andtback, Viveca Björnhagen-Säfwenberg, Weng-Onn Lui, Giuseppe V. Masucci, Lisa Villabona

###### Department of oncology/pathology, Karolinska Instituet, Stockholm, Sweden

*Journal of Translational Medicine* 2019, **17(Supp 1)**:11

**Background:** Merkel cell carcinoma (MCC) is a rare and aggressive neuroendocrine skin cancer which had an increasing incidence in the Swedish population (1). The pathogenesis is linked to the immune system and immunocompromised patients have an increased risk of developing MCC (2). With the discovery of Merkel cell polyoma virus (MCPyV), which is present in about 80% of MCC (3) the suggestion of a connection with the immune system has been strengthened. Previous reports (4) has shown that patients with a virus-negative disease have a worse prognosis. Furthermore, recent treatment with PD1 or PDL-1 blockade has shown to be successful in patients with advanced MCC (5, 6).

Our aim is to analyze clinical variables and their prognostic impact in a Swedish cohort of MCC-patients, in order to better understand possibilities to personalize treatment in the future.

**Methods:** 106 MCC patients referred to the plastic surgery unit at Karolinska University Hospital from 1989 to 2017 were included and retrospective data was collected. 65 (61%) were identified as female, 41 (39%) as male. Mean age at operation was 75.5 years, mean overall survival (OS) was 4.2 ± 0.5 years. MCPyV status was available in 42 patients, 5 of these were excluded due to lacking retrospective data. Out of the 37 patients, 24 (65%) patients were MCPyV positive, 13 (35%) MCPyV negative.

**Results:** Survival analysis did not show any significant difference between MCPyV positive and negative patients. When gender was added into multivariate analysis we found that female patients with virus negative disease had a significantly better outcome than virus negative male patients (p = 0.02) whereas virus positive MCC did not show any significant difference in OS between female and male patients.

**Conclusions:** Female patients with negative MCPyV MCC had a better outcome than the male patients. This finding indicates that MCPyV positive and negative MCC act as two different diseases. It also raises questions if there is a difference in the disease or immune response between male and female patients.

**References**

1. Zaar O, Gillstedt M, Lindelof B, Wennberg-Larko AM, Paoli J. Merkel cell carcinoma incidence is increasing in Sweden. J Eur Acad Dermatol Venereol. 2016;30(10):1708–13.
2. Engels EA, Frisch M, Goedert JJ, Biggar RJ, Miller RW. Merkel cell carcinoma and HIV infection. Lancet. 2002;359(9305):497–8.
3. Feng H, Shuda M, Chang Y, Moore PS. Clonal integration of a polyomavirus in human Merkel cell carcinoma. Science. 2008;319(5866):1096–100.
4. Moshiri AS, Doumani R, Yelistratova L, Blom A, Lachance K, Shinohara MM, et al. Polyomavirus-Negative Merkel Cell Carcinoma: A More Aggressive Subtype Based on Analysis of 282 Cases Using Multimodal Tumor Virus Detection. J Invest Dermatol. 2017;137(4):819–27.
5. Nghiem PT, Bhatia S, Lipson EJ, Kudchadkar RR, Miller NJ, Annamalai L, et al. PD-1 Blockade with Pembrolizumab in Advanced Merkel-Cell Carcinoma. N Engl J Med. 2016;374(26):2542–52.
6. Kaufman HL, Russell JS, Hamid O, Bhatia S, Terheyden P, D’Angelo SP, et al. Updated efficacy of avelumab in patients with previously treated metastatic Merkel cell carcinoma after >/=1 year of follow-up: JAVELIN Merkel 200, a phase 2 clinical trial. J Immunother Cancer. 2018;6(1):7

#### O12 Impact of BMI on clinical outcome in advanced cancer patients treated with anti-PD-1 Immune checkpoint inhibitors: a preliminary analysis

##### Alessio Cortellini^1^, Michele De Tursi^2^, Nicola Tinari^2^, Antonino Grassadonia^2^ Alessandro Parisi^1^, Laura Iezzi^2^, Pietro Di Marino^3^, Nicola D’Ostilio^4^, Davide Brocco^3^, Melissa Bersanelli^5^, Marta Peri^3^, Katia Cannita^1^, Corrado Ficorella^1^ and Clara Natoli^2^

###### ^1^Medical Oncology Unit, San Salvatore Hospital, Department of Biotechnological and Applied Clinical Sciences, University of L’Aquila, L’Aquila, Italy; ^2^Department of Medical, Oral and Biotechnological Sciences, CeSI-MeT, University G. D’Annunzio, Chieti, Italy; ^3^Clinical Oncology Unit, S.S. Annunziata Hospital, Chieti, Italy; ^4^Medical Oncology, San Pio da Pietralcina Hospital, Vasto, Italy; ^6^Medical Oncology Unit, University Hospital of Parma, Parma, Italy

*Journal of Translational Medicine* 2019, **17(Supp 1)**:12

**Background:** Interactions between nutrition and inflammation have been investigated and body mass index (BMI) has historically been considered the major surrogate of nutritional status.

**Patients and methods:** Baseline BMI was evaluated in advanced cancer patients consecutively treated with the anti-PD-1 Immune Check Point Inhibitors Nivolumab and Pembrolizumab monotherapy. Uni/multivariate analyses were performed to correlate BMI with clinical outcomes.

**Results:** Median age was 67.8 years (range 27–83). Primary tumors were: renal cell carcinoma (22 patients, 14.7%), melanoma (30 patients, 20%), NSCLC (90 patients, 60%), others (6 patients, 5.3%). Female/Male ratio was 45/105. 92 patients (61.3%) had an “high” BMI (≥ 25). All patients’ features are summarized in Table 1. Median follow-up was 9.9 months (range 1–38); median PFS was 7.9 months (95% CI 4.8–25.4; 48 events) among patients with BMI ≥ 25, and 3.9 months (95% CI 3.1–8.0; 35 events) among patients with BMI < 25. At multivariate analysis BMI < 25 was significantly related to a shorter PFS (HR = 1.73; 95% CI 1.09–2.74; p = 0.0184) (Table 2, Fig. 1). Survival data are still immature, with 96 censored patients at the data cut-off; the median OS was 12.4 months (95% CI 4.7–23.8) among patients with BMI < 25, while it was not-reached for patients with BMI ≥ 25; (HR = 1.57; 95% CI 0.92–2.70; p = 0.0974). No significant differences were observed in ORR and incidence of irAEs between the two subgroups.

**Table 1:** Patients’ characteristics

	No (%)
Screened patients	150
Age, (years)	
Median	67.8
Range	27–83
Elderly (≥ 70)	59
Sex	
Male	105 (70)
Female	45 (30)
ECOG PS	
0–1	114 (76)
≥ 2	36 (24)
Primary tumor	
NSCLC	90 (60)
Melanoma	30 (20)
Renal cell carcinoma	22 (14.7)
Others	8 (5.3)
No. of metastases	
≤ 2	47 (31.4)
> 2	103 (68.6)
Type of anti-PD-1	
Pembrolizumab	45 (30)
Nivolumab	105 (70)
Line of immunotherapy	
First	39 (26)
Non-first	111 (74)
irAEs of any grade	41 (27.3)
BMI (kg/m^2^)	
Median (range)	26 (16–45)
Underweight (BMI ≤ 18.5), no (%)	5 (3.3)
Normal weight (BMI 18.5 < BMI ≤ 24.9), no (%)	53 (35.3)
Overweight (25 < BMI ≤ 29.9), no (%)	57 (38)
Obese (BMI ≥ 30), no (%)	35 (23.4)

**Table 2:** Univariate and multivariate analyses for PFS

Variable (comparator)	Progression free survival			
	Univariate analysis		Multivariate analysis	
	HR (95% CI)	*p*-*value*	HR (95% CI)	*p*-*value*
BMI (< 25 vs ≥ 25)	1.71 (1.09–2.67)	*0.0182*	1.73 (1.09–2.74)	*0.0184*
Primary tumor (NSCLC)				
Melanoma	0.53 (0.29–0.99)	*0.0463*	0.48 (0.26–0.91)	*0.0236*
Kidney	0.75 (0.39–1.43)	*0.3880*	0.59 (0.31–1.48)	*0.1223*
Others	0.77 (0.27–2.13)	*0.6191*	0.80 (0.28–2.23)	*0.6715*
Sex (Male vs Female)	1.23 (0.76–1.99)	*0.3881*	1.15 (0.71–1.87)	*0.5734*
Age (Elderly vs Non-elderly)	1.04 (0.66–1.61)	*0.8570*	–	–
Treatment line (Non-first vs First)	1.72 (0.96–3.06)	*0.0639*	–	–
Burden of disease (> 2 vs ≤ 2 sites)	1.20 (0.75–1.92)	*0.4295*	–	–
ECOG PS (≥ 2 vs 0–1)	2.29 (1.86–4.70)	< *0.0001*	2.94 (1.84–4.69)	< *0.0001*

**Fig. 1:**
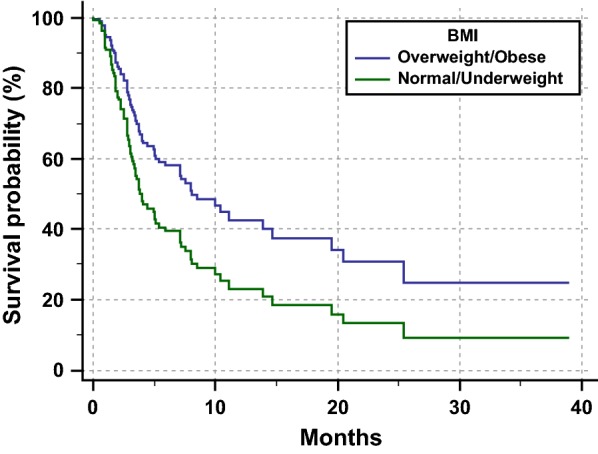
Multivariate analysis PFS, Cox proportional-hazards regression; p = 0.0184

**Conclusion:** It is known that nutritional status could affect immune responses: this preliminary report suggests a positive predictive impact of a high BMI on clinical outcomes of patients treated with anti-PD-1 ICIs. Further studies on the topic are required.

**Conflict of interest:** None.

**Funding sources:** None.

**Acknowledgements:** This work was supported by the Consorzio Interuniversitario Nazionale per la Bio-Oncologia (CINBO).

## Immunotherapy Bridge 2018

### Posters

#### P1 Changes of coding and non coding RNAs expression in extracellular vesicles (EVs) of patients with metastatic melanoma (MM) and treated with systemic therapy

##### Sonia Brugnara^1^, Elisabetta Grego^1,^ Viviana Anelli^2^, Vito D’Agostino^2^, Michela Notarangelo^2^, Michela Frisinghelli^1^, Mariacristina Sicher^4^, Calogero Pagliarello^4^, Carlo Renè Girardelli^4^, Paolo Cristofolini ^5^, Mario Cristofolini ^6^, Alessandro Quattrone^2^, Mattia Barbareschi^3^, Orazio Caffo^1^ e Marina Mione^2^

###### ^1^Oncology Unit S. Chiara Hospital, Trento, Italy; ^2^Center for Integrative Biology, University of Trento, Trento, Italy; ^3^Pathology Unit S. Chiara Hospital, Trento, Italy; ^4^Dermatology Unit S. Chiara Hospital, Trento, Italy; ^5^Plastic Surgery Unit S. Chiara Hospital, Trento, Italy; ^6^LILT Section, Trento, Italy

*Journal of Translational Medicine* 2019, **17(Supp 1)**:13

**Background:** There is a growing interest in assessing the expression of coding and non coding RNAs, due to their postulated regulatory role in DNA transcription. These nucleic acid fragments may be isolated in Extracellular Vesicles (EVs), which are found in the serum of patients with different types of tumors. An increased production of extracellular vesicles (EVs) was reported in metastatic melanoma (MM) (1,2). The present pilot study is aimed to assess RNAs expression in EVs extracted from serum of patients with MM treated with targeted therapy, immunotherapy or chemotherapy, to evaluate their role as biomarkers of response or resistance to the different therapies, and to validate a protocol for efficient study of the RNA content in EVs.

**Materials and methods:** We enrolled a consecutive series of 10 MM patients (stage M1a,b,c,d): 12 ml of whole blood was obtained from each patient at different timepoints: before starting therapy and during treatment (at 3 and 6 months from the treatment start). EVs were obtained from serum using a new isolation method (Notarangelo et al. submitted) developed in our laboratory. In addition, whole blood samples were obtained by 3 healthy donors. Total RNA in EVs was isolated, size fractionated and subjected to Next Generation Sequencing (NGS). We will evaluate the relationship between the changes of RNA expression and the response to the different treatments.

**Results:** The RNA analysis is still ongoing: at the time of the meeting we’ll present the isolation method and the results of the RNA expression changes according to the treatment responses.

**Conclusions:** This study will explore the role of RNA changes to predict the response to different therapies in MM, mainly by detecting primary or acquired resistance to targeted and immune therapy or chemotherapy. These tools could help to identify promptly the development of resistance leading to an early therapy switch.

**References**

1. Xiao et al., Identifying mRNA, microRNA and protein profiles of melanoma exosomes. PLoS One, 10: e46874 (2012);
2. Pfeffer et al., Detection of Exosomal miRNAs in the Plasma of Melanoma Patients J. Clin. Med. Res., 12:2012–27 (2015).

#### P2 Immune-related adverse events associated with checkpoint inhibitors therapy in a real world setting

##### Teresa Amaral, Thomas Eigentler, Paloma Caballero, Ioanna Tampouri, Diana Lomberg, Zeinab Assi, Ioannis Thomas, Ansgar Koechel, Alisa Mueller, Lukas Kofler, Ulrike Leiter, Andrea Forschner, Claus Garbe

###### Center for Dermatooncology, Department of Dermatology, Liebermeisterstrasse. 25, University Hospital Tuebingen, Tuebingen, Germany

*Journal of Translational Medicine* 2019, **17(Supp 1)**:14

**Background:** Adverse events (AE) associated with immunotherapy (IT) are becoming more frequent in the clinical practice, as checkpoint inhibitors are currently approved in several indications.

**Patients and Methods**: We conducted a retrospective analysis of patients’ diagnosed with melanoma and non-melanoma skin cancers and treated with IT between January 2015 and December 2016 in the University Hospital Tuebingen. The following information regarding immune-related AE (irAEs) was documented: organ involved, frequency, time of onset after immunotherapy initiation, duration, and outcome. irAEs were classified using CTCAE term and MedDRA SOC classification (version 4.0). Only patients who developed a toxicity grade ≥ 2 were assigned to the group of patients having AE.

**Results**: From 256 patients treated with IT, 88 patients developed 140 irAEs [38 females, 50 males; median age 64 years old (min: 39 years; max: 87 years)].

The irAEs were associated with ipilimumab + nivolumab in 60 cases, with pembrolizumab in 46 cases, with nivolumab in 18 cases and with ipilimumab in 16 cases. More detailed information is available in Table 1.

**Table 1:** irAEs frequency and time of onset after IT initiation, considering the MedDRA SOC classification

irAEs	Frequency (n = 140)	Median time to onset (weeks)	Min–max time to onset (weeks)
Gastrointestinal	36	13	1–169
Endocrine	28	19	1–108
Hepatobiliary	19	6	0–30
Respiratory	13	22	1–78
Others	44	16	1–84

Hospitalization was required for almost half of the cases and the majority of the irAEs persisted for more than 4 weeks (50%). Corticotherapy was used in 55% of the irAEs. Excluding endocrine irAEs, which didn’t recover completely, a full recovery was observed in 43% of the cases.

**Conclusion**: Severe irAEs are frequent in patients treated with IT in the daily practice. These can occur as soon as 1 week after initiating therapy but also much later—up to 3 years in our collective. This highlights the need to thoroughly inform this population before, during and after receiving IT. Although most of the irAEs responded to steroid administration and temporary treatment suspension, 50% lasted for more than 4 weeks and hospitalization was required in a high percentage of cases, increasing the costs associated with AE management.

#### P3 A retrospective study of adverse events of immunotherapeutic drugs, Nivolumab, Ipilimumab and Pembrolizumab, reported in Italy, from 2015 to 2017

##### Antonio D’Avino, Roberta D’Aniello, Piera Maiolino

###### S.C. Farmacia, IRCCS Fondazione G. Pascale, Napoli, Italy

*Journal of Translational Medicine* 2019, **17(Supp 1)**:15

**Background:** Immunotherapies, like ipilimumab, nivolumab, and pembrolizumab, have changed the cancer treatment landscape. They are inhibitors of protein, which cancer cells use to elude the immune system. These monoclonal-antibody are recently approved by the Italian Medicines Agency for therapy of melanoma.

Post-marketing surveillance of these drugs have revealed severe and immune-mediate adverse drug reactions (ADRs).

Occurrence of ADRs has high morbidity and mortality. Italian Medicines Agency (AIFA) uses the web-based monitoring system, National Network of Pharmacovigilance (RNF) for the reporting and evaluation of suspicious ADRs, reported, with national detection, loaded on the system RNF national web.

**Aim:** Retrospective observational study was done. Aim of the study is to investigate the ADRs occurred in patients, in Italy, treated with PD-1 (Nivolumab, Pembrolizumab) and CTLA-4 (Ipilimumab) inhibitors and uploaded to RNF, reported from the years 2015 to 2017.

**Methods:** All ADRs associated with Nivolumab, Ipilimumab and Pembrolizumab reported in the RNF, with national detection, during the period 2015–2017, were analysed for overall numbers, age (y), gender, serious (severe-ADRs) and immune-mediated (immune-ADRs) adverse event. Quantitative measurement [proportional reporting ratio (PRRs)] was used to summarize the extent to which a severe-ADRs, or immune-ADRs, is reported for individuals taking a specific drug, compared to the frequency at which the same adverse event is reported for patients taking the other two drugs.

**Results:** 1288 ADRs have been reported and analysed in this study. Nivolumab (82.7%), Pembrolizumab (4.2%) and Ipilimumab (12.8%). 64.8% occurred in males (male/female ratio = 2.09). Most ADRs occurred in patients with age over 60 years (70.2%). The higher incidence of ADRs occurred in severe events 45% vs 2.2% that was represent by immune-mediate adverse event.

Severe-ADRs are the most reported ADRs category for Nivolumab and Ipilimumab (36% vs 6.3%) respectively 464 and 82. Pembrolizumab^®^ (34severe-ADRs) shows a PRR = 1.2 for treated patients, than Ipilimumab and Nivolumab treated patients.

Immune-ADRs are the most reported ADRs category for Ipilimumab^®^ (18 ADRs) with a PRR = 10 for treated patients, than Pembrolizumab and Nivolumab treated patients.

**Conclusion:** Analysis of ADRs, is important to understand the safety of drugs in post-marketing and in real clinical practice. By this study is evident that between all ADRs reported from the years 2015 to 2017, immune-mediated event shown 10 times more for Ipilimumab than Nivolumab and Pembrolizumab, and severe event shown 1, 2 times more for Pembrolizumab than Nivolumab and Ipilimumab. Limitations of this study was cases were based on spontaneous reporting which clearly suffered from underreporting despite the analyzed dates estimates plausible values. Clinical data were not available.

## Immunotherapy Bridge 2018

### Author keynotes

#### K1 Systems immunology and tumor microenvironment: novel perspectives

##### Jérôme Galon

###### ^1^Director of Research, French National Institute of the Health and Medical Research (INSERM), Chief of laboratory of Integrative Cancer Immunology, Cordeliers Research Center, Paris, France

*Journal of Translational Medicine* 2019, **17(Supp 1)**:15.1

To date, the anatomic extent of tumor (TNM-classification) has been by far the most important factor to evaluate the prognosis of cancer patients. However, this classification provides limited prognostic information and does not predict response to therapy. We redefined cancer by integrating the immune system, to transfer cutting-edge medicine to the patients. We have previously shown that tumors from human colorectal cancer with a high-density of infiltrating memory and effector-memory T-cells (Tem) are less likely to disseminate to lymphovascular and perineural structures and to regional lymph-nodes. We also demonstrated the critical tumor-microenvironment parameters determining the dissemination to distant metastasis. We found that the combination of immune parameters associating the nature, the density, the functional immune orientation and the location of immune cells within the tumor was essential to accurately define the impact of the local host-immune reaction on patients’ prognosis. We defined these parameters as the “immune contexture”. We characterized the immune landscape within human tumors, and showed the importance of adaptive immune cells including, cytotoxic T-cells, Th1-cells, B-cells and T-follicular-helper (Tfh) cells. We described the immunophenotype and antigenome associated with immune escape mechanisms and demonstrated mechanisms associated with pre-existing and proliferating intratumoral T-cells.

Based on the *immune contexture*, a standardized, simple and powerful digital-pathology-based immune stratification-system, termed *“Immunoscore”*, was delineated having a prognostic power superior to that of the currently used cancer staging-system. Tumor invasion parameters were statistically dependent on the host-immune reaction. A worldwide consortium validated the prognostic value of the consensus *Immunoscore*, using a standardized-assay.

Very recently, we conducted comprehensive analyses revealing a large inter and intra-metastatic immune heterogeneity. Nonetheless, even when measured on a single biopsy, the *Immunoscore* held a prognostic value and was surpassing the accuracy of PD-L1 expression. Our most recent data supported the significant role of *Immunoscore* and immunoediting in affecting metastatic dissemination. We hence proposed a “parallel selection model” of tumor evolution incorporating the effects of the immune system in shaping and driving metastatic spread. Thus, tumor progression, invasion and recurrence are dependent on pre-existing immunity and on *Immunoscore*.

## Melanoma Bridge 2018

### Melanoma as a model system session—oral communications

#### O13 Treatment patterns and overall survival outcomes among *BRAF* wild-type metastatic melanoma patients receiving first-line anti-programmed cell death protein-1 therapy in routine clinical practice in the United States

##### Natalia Sadetsky^1^, Ching-Yi (James) Chuo^1^, Edward McKenna^1^, Sarah Troutman^1^, Dawn Colburn^1^, Michael B. Atkins^2^

###### ^1^PHC Data Science, Genentech, Inc., South San Francisco, CA, USA; ^2^Department of Medical Oncology, Georgetown University, Washington, DC, USA

*Journal of Translational Medicine* 2019, **17(Supp 1)**:16

**Background:** Despite recent advances in first-line therapy for patients with *BRAF* wild-type (wt) metastatic melanoma, post-progression treatment options are limited. Our aim was to evaluate treatment patterns and overall survival (OS) in patients with *BRAF*wt metastatic melanoma receiving first-line anti-programmed cell death protein-1 (aPD-1) in routine clinical practice in the United States.

**Materials and methods:** Patients with *BRAF*wt metastatic melanoma receiving first-line aPD-1 therapy (pembrolizumab [PEM], nivolumab [NIVO], or ipilimumab [IPI] + NIVO) between 1/1/2014 and 11/30/2017 were identified in the Flatiron Health Database (Flatiron Health Data, New York, NY). Patient status was defined as (1) deceased; (2) alive, continuing or having completed first-line ≤ 30 days of data cutoff with no evidence of subsequent treatment (second-line); or (3) alive with evidence of second-line therapy.

**Results:** Of 434 *BRAF*wt patients, 167 (38.5%) received PEM, 136 (31.3%) received IPI + NIVO, and 131 (30.2%) received NIVO. At data cutoff (11/30/2017), 146 patients (33.6%) had died. Of 288 patients still alive, 208 patients (72%) continued or completed first-line therapy, and 80 patients (28%) had received second-line therapy. Median OS for patients receiving first-line aPD-1 therapy was 21.1 (16.7–NA) months and varied significantly by baseline lactate dehydrogenase (LDH) level (LDH low-normal: not reached [19.45–NA] months and LDH high: 16.8 [6.2–NA] months). Median OS for patients with second-line therapy was 8.1 months, and similar differences in survival outcomes by baseline LDH levels were observed. The most common reasons for discontinuing first-line therapy included disease progression (41 patients [51%]) and treatment toxicity (11 patients [14%]). Among patients who underwent second-line therapy, cancer immunotherapy (CIT) and chemotherapy alone or in combination with CIT were used by 51 patients (64%) and 27 patients (34%), respectively.

**Conclusions:** This real-world study evaluating treatment utilization and outcomes in *BRAF*wt patients exposed to aPD-1 therapy in first-line demonstrated limited second-line treatment options and survival benefit, highlighting the high unmet need for new therapeutic options in this patient population.

#### O14 Major pathologic response on biopsy (MPR_bx_) in patients with melanoma treated with anti-PD-1: evidence for an early, on-therapy biomarker of response

##### Julie E. Stein^1^, Abha Soni^1^, Ludmila Danilova^3^, Tricia R. Cottrell^2^, William H. Sharfman^4^, Megan Wind-Rotolo^5^, Robin Edwards^5^, Evan J. Lipson^4^, Janis M. Taube^1,2,4^

###### ^1^Departments of Dermatology at Johns Hopkins University SOM, the Sidney Kimmel Comprehensive Cancer Center, and the Bloomberg–Kimmel Institute for Cancer Immunotherapy at Johns Hopkins, Baltimore, Maryland, USA, ^2^Departments of Pathology at Johns Hopkins University SOM, the Sidney Kimmel Comprehensive Cancer Center, and the Bloomberg–Kimmel Institute for Cancer Immunotherapy at Johns Hopkins, Baltimore, Maryland, USA, ^3^Departments of Biostatistics at Johns Hopkins University SOM, the Sidney Kimmel Comprehensive Cancer Center, and the Bloomberg–Kimmel Institute for Cancer Immunotherapy at Johns Hopkins, Baltimore, Maryland, USA, ^4^Departments of Oncology at Johns Hopkins University SOM, the Sidney Kimmel Comprehensive Cancer Center, and the Bloomberg–Kimmel Institute for Cancer Immunotherapy at Johns Hopkins, Baltimore, Maryland, USA; ^5^Bristol-Myers Squibb, Princeton, New Jersey, USA

*Journal of Translational Medicine* 2019, **17(Supp 1)**:17

**Background:** With the increasing use of anti-PD-1 in patients with melanoma and other tumor types, there is great interest in developing an early on-treatment biomarker that correlates with long-term patient outcomes. An understanding of the pathologic features of immune-mediated tumor regression is key in this endeavor. The objective of this study was to identify the features of anti-PD-1-associated immune-mediated melanoma regression and correlate them with patient outcomes.

**Materials and methods:** We assessed hematoxylin and eosin (H&E)-stained slides, from formalin-fixed paraffin-embedded (FFPE) tissue, for pathologic features in N = 139 pre- and on-treatment melanoma specimens from 79 unique patients treated with anti-PD-1. Response to therapy was assessed using RECIST v1.1. The discovery cohort consisted of archival specimens (n = 30) from patients with advanced melanoma treated at Johns Hopkins; validation cohort specimens (n = 109) originated from patients enrolled on the nivolumab monotherapy arm of CA209-038 (NCT01621490). Immune and non-immune pathologic features such as lymphoid aggregates, neovascularization, plasma cells, proliferative fibrosis, necrosis, and dense collections of melanophages were studied. Tumor infiltrating lymphocyte (TIL) density was also scored. Pathologic features were compared between pre- and on- anti-PD-1 therapy specimens and between responders and non-responders, and a constellation of immune-related pathologic response (irPR) features was identified. These were used to develop an irPR score (from 0 = no irPR features to 3 = major pathologic response on biopsy (MPR_bx_, ≤ 10% residual viable tumor). In the validation cohort, irPR scores were evaluated for association with patient outcome.

**Results:** In the discovery cohort, on-treatment specimens from responders (n = 7) had features of immune-activation (moderate-high TIL densities, plasma cells) and wound-healing/tissue repair (neovascularization, proliferative fibrosis) compared to non-responders (n = 7), (p ≤ 0.021, for each feature). In the validation cohort (n = 14 responders and n = 19 non-responders), increasing irPR scores associated with objective response to anti-PD-1 (p = 0.014) and MPR_bx_ was associated with increased overall survival (n = 51; HR 0.13; 95% CI 0.054–0.31, p = 0.015). Neither tumoral necrosis nor pre-treatment histologic features were associated with response. Eight of 16 (50%) of patients with stable disease showed irPR features.

**Conclusions:** We have identified histopathologic features of immune-mediated tumor regression on routine H&E-stained slides in patients with melanoma that correlate with objective response to anti-PD-1 and OS. This approach is inexpensive and widely available. Our findings inform grading systems for pathologic response following administration of checkpoint blocking agents and will likely influence future biomarker strategies.

#### O15 Immune monitoring after NKTR-214 plus nivolumab (PIVOT-02) in previously untreated patients with metastatic Stage IV melanoma

##### Adi Diab^1^, Scott Tykodi^2^, Brendan Curti^3^, Daniel Cho^4^, Mike Wong^1^, Igor Puzanov^5^, Karl Lewis^6^, Michele Maio^7^, Gregory A. Daniels^8^, Alexander Spira^9^, Mary Tagliaferri^10^, Alison Hannah^10^, Wendy Clemens^10^, Michael Imperiale^10^, Chantale Bernatchez^1^, Cara Haymaker^1^, Salah Eddine Bentebibel^1^, Jonathan Zalevsky^10^, Ute Hoch^10^, Christie Fanton^10^, Ahsan Rizwan^10^, Sandra Aung^10^, Fiore Cattaruzza^10^, Ernesto Iaccucci^10^, Dariusz Sawka^11^, Mehmet Bilen^12^, Paul Lorigan^13^, Giovanni Grignani^14^, James Larkin^15^, Sekwon Jang^16^, Ewa Kalinka Warzocha^17^, Mario Sznol^18^, Mike Hurwitz^18^

###### ^1^The University of Texas MD Anderson Cancer Center, Houston, TX, USA; ^2^University of Washington and Fred Hutchinson Cancer Research Center, Seattle, WA, USA; ^3^Providence Cancer Institute and Earle A. Chiles Research Institute, Portland, OR, USA; ^4^NYU Medical Oncology Associates, New York, NY, USA; ^5^Roswell Park Cancer Institute, Buffalo, NY, USA; ^6^University of Colorado Denver, Denver, CO, USA; ^7^Azienda Ospedaliera Universitaria Senese, Italy; ^8^Moores Cancer Center, University of California San Diego, San Diego, CA, USA; ^9^Virginia Cancer Specialists, PC, Fairfax, VA, USA; ^10^Nektar Therapeutics, San Francisco, CA, USA; ^11^Szpital Specjalistyczny w Brzozowie Podkarpacki Osrodek Onkologiczny, Poland; ^12^Emory University Hospital (Winship Cancer Institute), Atlanta, GA, USA; ^13^The Christie NHS Foundation Trust, United Kingdom; ^14^Institute for Cancer Research and Treatment (IRCC), Italy; ^15^The Royal Marsden, United Kingdom; ^16^Inova Schar Cancer Institute, Fairfax, VA, USA; ^17^Instytut Medyczny Santa Familia, Poland; ^18^Yale School of Medicine, New Haven, CT, USA

*Journal of Translational Medicine* 2019, **17(Supp 1)**:18

**Background:** In patients with melanoma, low levels of tumor-infiltrating lymphocytes and low/absent PD-L1 expression are associated with limited response to anti-PD-1/anti-PD-L1 therapies. NKTR-214 (IL-2Rβγ-biased cytokine) monotherapy stimulates proliferation and activation of lymphocytes in blood and tumor and increases PD-1/PD-L1 expression. The impact of NKTR-214 plus nivolumab on the systemic immune system and local tumor microenvironment in the melanoma cohort of the PIVOT-02 phase 1/2 open-label study is presented.

**Methods:** Patients received NKTR-214 (0.003, 0.006 or 0.009 mg/kg) with nivolumab (240 or 360 mg) Q2 W or Q3 W (phase 1), and NKTR-214 (0.006 mg/kg) with nivolumab (360 mg) Q3 W (RP2D). Tumor biopsies were analyzed using multispectral IHC, gene expression, and TCR sequencing. Blood cells were evaluated using flow cytometry and hematology. PD-L1 expression was evaluated using DAKO, 28-8 PharmDx Assay.

**Results:** The melanoma cohort is closed; 41 patients were enrolled with 38 evaluable for efficacy (≥ 1 follow-up scan). Immune monitoring of blood showed clear activation of the IL-2 pathway following administration of NKTR-214 plus nivolumab. Lymphocyte numbers increased 9× (n = 41) from nadir reaching their peak 7 days postdose and maintained that magnitude of increase after each cycle. The proportion of proliferating (Ki67^+^, n = 12) CD4^+^, CD8^+^ and NK cells increased 13×, 20×, and 6× over baseline, respectively. Similar immune activation was reported with NKTR-214 monotherapy (8×, 8×, and 7× over baseline, respectively). Immune cells showed an antigen-experienced phenotype with increased proportion of HLA-DR expression on CD4^+^, CD8^+^, and NK cells 3×, 2×, and 6× over baseline, respectively. ICOS levels increased 2× on CD8^+^ T cells. Baseline and week 3 biopsies (n = 12, evaluable) demonstrated local effects on the tumor microenvironment including elevated expression of PD-L1 on the tumor (patients converted from PD-L1 negative to positive), increased total numbers of CD8 infiltrate, and increased proportion of proliferating cells all ranging from 6 to 17× over baseline. Following treatment, intratumoral gene expression analyses showed elevations in networks associated with the NKTR-214 mechanism of action, including induction of an interferon gamma-gene signature. The investigator-assessed ORR as of 12 July 2018 was 50% (n = 38), and no responder has relapsed. The median duration of response has not been reached.

**Conclusions:** NKTR-214 is a robust agonist of the IL-2 pathway and together with nivolumab promotes immune activation in the periphery and tumor microenvironment for significant clinical activity. A global phase 3 trial of NKTR-214 plus nivolumab versus nivolumab (1:1) in patients with treatment-naïve advanced melanoma opens for enrollment in Q3 2018.

Clinicaltrials.gov NCT02983045 (PIVOT-02).

## Melanoma Bridge 2018

### Mechanism of resistance and drivers of response session—oral communications

#### O16 Anti-PD-1 immunotherapy modulates PD-L1 expression on neutrophil subsets and monocytes from advanced melanoma patients

##### M. R. Galdiero^1,2,3^, L. Cristinziano^1,2,3^, M. Capone^4^, G. Madonna^4^, D. Mallardo^4^, S. Loffredo^1,2,3^, A. L. Ferrara^1,2,3^, M. A. Braile^1,2,3^, Vito Vanella^4^, Lucia Festino^4^, P.A. Ascierto^4^, G. Marone^1,2,3^

###### ^1^Department of Translational Medical Sciences (DiSMeT), ^2^Center for Basic and Clinical Immunology Research (CISI) ^3^WAO Center of Excellence, University of Naples Federico II, 80131 Naples, Italy; ^4^Melanoma Cancer Immunotherapy and Innovative Therapy Unit, Istituto Nazionale Tumori Fondazione “G. Pascale”, 80131 Naples, Italy

*Journal of Translational Medicine* 2019, **17(Supp 1)**:19

**Introduction:** Advanced melanoma is a life-threatening cancer with a median survival of 6–9 months. Monoclonal antibodies (mAbs) that disrupt programmed death (PD-1) and PD-Ligand 1 (PD-L1) have revolutioned cancer immunotherapy. PD-L1 is expressed on several immune cells and recent evidence indicates that can be also expressed on human neutrophils. In addition to Normal Density Neutrophils (NDNs), a population of “Low Density” neutrophils (LDNs) increases in chronic inflammatory conditions and correlates with cancer progression. The role of peripheral blood neutrophils and monocytes as predictive biomarkers in anti-PD-1 therapy response is largely unknown.

**Methods:** 39 Patients with advanced melanoma were prospectively recruited. PMNs and mononuclear cells were isolated from peripheral blood of healthy controls (HC) and melanoma patients, before and during anti-PD-1 therapy, to evaluate activation markers, PD-L1 expression, morphology, ROS production.

**Results:** NDNs from melanoma patients displayed increased activation markers and PD-L1 levels compared to HC, which reverted during anti-PD-1 immunotherapy. Melanoma patients presented increased number of LDNs compared to HC but their percentages did not change during immunotherapy. Patients LDNs displayed increased PD-L1 expression compared to autologous NDNs which dropped after 3 months of therapy. PD-L1 expressing monocytes were increased in patients and decreased after 3 months of therapy. Patients NDNs showed reduced ROS production and peculiar morphological aspects.

**Conclusions:** We found increased PD-L1 expression on peripheral blood NDNs, LDNs and monocytes in advanced melanoma patients, which was modulated by anti-PD-1 immunotherapy. Ongoing investigations are evaluating whether PD-L1 expressing myeloid cells can be associated with patient clinical outcome.

#### O17 Mechanisms of primary resistance to immune checkpoint inhibitors in Melanoma

##### Duane Moogk^1^, Lin Wang^1^, Kaitao Li^2^, Zhou Yuan^3^, Shi Zhong^1^, Zhiya Yu^4^, Ivan Liadi^5^, William Rittase^3^, Victoria Fang^1^, Janna Dougherty^1^, Arianne Perez-Garcia^1^,, Navin Varadarajan^5^, Nicholas P. Restifo^4^, Alan Frey^6^, Iman Osman^1,7^, Jeff Weber^1,8^, Cheng Zhu^2,3^, Michelle Krogsgaard^1,9^

###### ^1^Perlmutter Cancer Center, Departments of Cell Biology, New York University School of Medicine; ^2^Coulter Department of Biomedical Engineering, Georgia Institute of Technology; ^3^George W. Woodruff School of Mechanical Engineering, Georgia Institute of Technology; ^4^Center for Cancer Research, NCI, NIH; ^5^Department of Chemical and Biomolecular Engineering, University of Houston; ^6^Perlmutter Cancer Center, Departments of Cell Biology, New York University School of Medicine; ^7^Departments of Dermatology, New York University School of Medicine; ^8^Departments of Medicine, New York University School of Medicine; ^9^Departments of Pathology, New York University School of Medicine

*Journal of Translational Medicine* 2019, **17(Supp 1)**:20

**Background:** Although much clinical progress has been made in harnessing the immune system to recognize and target cancer, there is still a significant lack of an understanding of how tumors evade immune recognition and the mechanisms that drive tumor resistance to both T cell and checkpoint blockade immunotherapy. Our objective is to understand how tumor-mediated signaling through multiple inhibitory receptors, including PD-1, combine to affect the process of T cell recognition of tumor antigen and activation signaling. This with the goal of understanding the basis of resistance to PD-1 blockade and potentially identify new molecular targets to enable T cells to overcome dysfunction mediated by multiple inhibitory receptors.

**Methods and Results:** Biomembrane Force Probe *(BFP)* measurements show that that the activities of TCR-proximal signaling components affect T cell mechanosensing and sensitivity at the earliest stages of antigen recognition and are influenced by PD-1 and other inhibitory receptors via Shp-1/2 by targeting CD28 and Lck to directly suppress TCR-pMHC-CD8 binding. Phospho-proteomics and flow cytometry-based analysis of patient-derived T cells from PD-1 responders and non-responders identified additional mediators, signaling components and pathways associated with PD-1 checkpoint blockade resistance. CRISPR/Cas9 mediated genome editing was utilized to determine if resistance is mediated by the continued signaling of multiple IRs by perturbing IR signaling in mouse models of PD-1 blockade.

**Conclusions:** Our results suggest that T cell activation signaling and effector function is altered in PD-1 blockade-resistant patients due to the combined signaling of multiple inhibitory receptors, and therefore clinical targeting of pathways common to multiple inhibitory receptors can overcome PD-1 blockade resistance. Targeting these interactions and understanding the basis of resistance to PD-1 blockade would potentially allow identification of novel biomarkers of resistance or new molecular targets to enable T cells to overcome dysfunction during PD-1 checkpoint blockade and improve patient outcomes.

**Acknowledgement:** This work was supported by research grants from National Institute of Health U01 CA214354 (to M.K. and C.Z.) and Merck OTSP grant 58166 (to M.K.) and 57570 (to M.K.).

**References**

1. D. Moogk et al., “Constitutive Lck activity drives sensitivity differences between CD8 + memory T cell subsets,” *J. Immunol.,* 15;197(2):644–54, July 2016.
2. E. Hui et al., “T cell costimulatory receptor CD28 is a primary target for PD-1-mediated inhibition,” *Science,* vol. 355, no. 6332, pp. 1428–1433, Mar 31 2017.
3. J. Casas et al., “Ligand-engaged TCR is triggered by Lck not associated with CD8 coreceptor,” *Nat Commun,* vol. 5, p. 5624, 2014.
4. N. Jiang et al., “Two-stage cooperative T cell receptor-peptide major histocompatibility complex-CD8 trimolecular interactions amplify antigen discrimination,” *Immunity,* vol. 34, no. 1, pp. 13–23, 2011.
5. E. Evans, K. Ritchie, and R. Merkel, “Sensitive force technique to probe molecular adhesion and structural linkages at biological interfaces,” *Biophys J,* vol. 68, no. 6, pp. 2580–7, Jun 1995.
6. J. Huang et al., “The kinetics of two-dimensional TCR and pMHC interactions determine T cell responsiveness,” *Nature,* vol. 464, no. 7290, pp. 932–6, Apr 8 2010.

#### O18 Intratumoral tavokinogene telseplasmid induces abscopal clinical responses in metastatic melanoma patients

##### Alain Algazi^1^, Shailender Bhatia^6^, Katy K. Tsai^1^, Sharron Gargosky^2^ Donna Bannavong^2^, Reneta Talia^2^, Erica Browning^2^, Christopher G. Twitty^2,^ David Canton^2^, Jean Campbell^2^, Robert H. Pierce^2^, Mai H. Le^2^, Carmen Ballesteros-Merino^3^, Carlo B. Bifulco^3^, Bernard A. Fox^3^, Mark Faries^4^, Manuel Molina^5^, Sanjiv Agarwala^7^, Karl Lewis^8^, and Adil Daud^1^

###### ^1^University of California San Francisco, San Francisco, California, USA; ^2^OncoSec Medical Incorporated, San Diego, California, USA; ^3^Earle A. Chiles Research Institute at Providence Portland Medical Center Portland, Oregon, USA; ^4^John Wayne, Los Angeles, California, USA; ^5^Lakeland Health Medical Center, Lakeland, Florida, USA; ^6^University of Washington, Seattle, Washington, USA; ^7^St. Luke’s Cancer Center, Bethlehem, Pennsylvania, USA; ^8^University of Colorado Cancer Center, Aurora, Colorado, USA

*Journal of Translational Medicine* 2019, **17(Supp 1)**:21

The combination of tavokinogene telseplasmid (plasmid IL-12 or “tavo”) and pembrolizumab has been shown to induce frequent, durable clinical responses in patients with immunologically “cold” melanoma. To assess the unique contribution of intratumoral (IT) tavo to systemic anti-tumor immune responses, we examined regression of untreated lesions, the hallmark of systemic response to IT immunotherapy, in patients treated with IT-tavo monotherapy. Stage III/IV melanoma patients were enrolled in a phase 2 trial where 1–4 accessible lesions were treated and at least one lesion was left untreated (NCT01502293). The sums of the diameters of treated and untreated lesions were assessed by modified RECIST (allows tumors > 0.3 cm and more lesions/organ). AEs were assessed by CTCAE version 4. Paired biopsies were assessed for PD-L1 levels by IHC and for changes in inflammatory gene expression. 51 patients were enrolled. 35 patients (69%) had been previously treated with systemic therapy (22 with 2 or more therapies). The most common AEs were pain and local reactions at treatment sites. There were no grade 4 or 5 AEs during the study. Two patients (3.9%, 2/51) had AEs leading to study withdrawal. Best overall response was 29.2% (10.4% CR [5/48 patients] and 18.8% PR [9/48 patients]). Time to objective response (min–max) was 0.9–3.9 months. The median duration of response was 7.3 months and the median time to progression was 2.7 months. The median OS was not met at median follow-up of 28 months. Longitudinal response assessment suggested 3 response categories: durable, transient, and no response. This clinical data demonstrates that IT-tavo can trigger potent systemic immune activation, evidenced by regression in 40% patients (16% of all untreated lesions) which dovetails with our previous paired tumor analysis highlighting that IT-tavo-EP increases TILs (both CD8 + T cells and NK cells) as well as intratumoral expression of interferon gamma associated genes. Our current data suggests that while this treatment-related abscopal effect and related increase of intratumoral inflammation can have a systemic impact on tumor burden, it also triggers adaptive resistance, manifested as increased PD-L1 expression, a major factor that could limit the clinical benefits of IT-tavo-EP monotherapy in some patients. This provides a strong rationale for combination with an anti-PD-1 therapy. In the context of our previously reported findings, our data also supports the hypothesis that anti-PD-1 Ab therapy amplifies this newly inflamed tumor microenviroment leading to durable, systemic responses in patients who would not otherwise benefit from PD-1 Ab monotherapy.

#### O19 A KDR germline variant is associated with increased risk of melanoma, a pro-angiogenic phenotype and resistance to immunotherapy

##### Irineu Illa-Bochaca^1^, Keith Giles^1^, Farbod Darvishian^2^, Una Moran^1^, Judy Zhong^3^, Michelle Krogsgaard^2^, Tomas Kirchhoff^4^, Iman Osman^1^

###### ^1^The Ronald O. Perelman Department of Dermatology, New York University School of Medicine, New York, NY, USA; ^2^Department of Pathology, NYU School of Medicine, New York, New York, USA; ^3^Department of Population Health, New York University School of Medicine, New York, New York, USA; ^4^Division of Epidemiology, New York University School of Medicine, New York, New York, USA

*Journal of Translational Medicine* 2019, **17(Supp 1)**:22

**Background:** Immune checkpoint inhibition, such as anti-CTLA-4 and anti-PD-1 blockade, significantly improve survival of melanoma patients, however, a substantial proportion of patients do not respond or develop resistance. As tumor angiogenesis has been reported to be associated with reduced anti-tumor immunity, we reasoned that a kinase insert domain receptor (KDR) germline variant (Q472H) is a driver of melanoma angiogenesis and might determine the clinical response to anti-CTLA-4 and/or anti-PD-1 therapy.

**Methods:** Germline DNA from 1429 stages I-IV melanoma patients enrolled in the NYU Interdisciplinary Melanoma Cooperative Group (IMCG) between 2010 and 2017 was genotyped to determine KDR variant status (MassARRAY iPLEX platform, Agena Bioscience). Clinical response was assessed for a cohort of melanoma patients (n = 206) treated with anti-CTLA-4 or anti-PD-1. Tumor angiogenesis was assessed by immunostaining with anti-CD34 antibody (Abcam) and quantitation of microvessel density (MVD) in melanoma tissues (n = 161).

**Results:** The KDR Q472 variant allele was found in 37% (536/1429) of melanoma patients, which is significantly higher (p = 0.001) than its frequency of 21% in the general population and 13% in Caucasians (1000 Genomes Project database [http://www.internationalgenome.org/]). Melanoma patients harboring germline KDR Q472H had significantly higher MVD (p = 0.04). Presence of KDR Q472H was not associated with response to anti-CTLA-4 (p = 0.8), but was associated with resistance to anti-PD-1 treatment (p = 0.02).

**Conclusions:** Our results suggest that the germline variant KDR Q472H is enriched in melanoma patients compared to general population, and promotes an angiogenic tumor phenotype, which might contribute in part to resistance to anti-PD-1 treatment in the metastatic setting. The lack of association with anti-CTLA-4 treatment might be due to the different mechanisms of action as CTLA-4 is thought to regulate T-cell proliferation early in the immune response, mostly in lymph nodes, whereas PD-1 suppresses T cells later in the immune response, primarily in peripheral tumor tissue where angiogenesis might have more of an effect.

#### O20 Efficacy of novel melanoma treatments in metastatic melanoma patients with germline CDKN2A mutations

##### Hildur Helgadottir^1^, Paola Ghiorzo^2^, Remco van Doorn^3^, Susana Puig^4,5^, Max Levin^6^, Richard Kefford^7^, Paola Queirolo^8^, Lorenza Pastorino^2^, Martin Lauss^9^, Håkan Olsson^9^, Veronica Höiom^1^, Göran Jönsson^9^

###### Department of Oncology Pathology, Karolinska Institutet and Karolinska University Hospital Solna, 171 76, Stockholm, Sweden; ^2^Department of Internal Medicine and Medical Specialties, University of Genoa and Genetics of Rare Cancers, Ospedale Policlinico San Martino, Genoa, Italy; ^3^Department of Dermatology, LUMC, Leiden, The Netherlands; ^4^Melanoma Unit, Dermatology Department, Hospital Clinic de Barcelona, Institut d’Investigacions Biomèdiques August Pi i Sunyer (IDIBAPS), Universitat de Barcelona, Barcelona, Spain; ^5^Centro de Investigación Biomédica en Red (CIBER) de Enfermedades Raras, Instituto de Salud Carlos III, Barcelona, Spain; ^6^Department of Oncology, The Sahlgrenska Academy, University of Gothenburg, Göteborg, Sweden; ^7^Westmead Hospital and Macquarie University, Sydney, New South Wales, Australia; ^8^Department of Medical Oncology, IRCCS Ospedale Policlinico San Martino, Genoa, Italy; ^9^Department of Oncology, Clinical Sciences Lund, Lund University and Skåne University Hospital, Lund, Sweden

*Journal of Translational Medicine* 2019, **17(Supp 1)**:23

Efficacy of novel melanoma treatments in metastatic melanoma patients with germline CDKN2A mutations.

**Background**: Somatic mutations and deletions in the CDKN2A gene are frequent driver events in melanoma tumors. Germline CDKN2A mutation is one of the strongest known risk factors for cutaneous melanoma. Individuals that carry inherited CDKN2A mutations are extremely prone to develop melanomas and mutation carriers are also reported to have inferior melanoma-specific survival.

**Methods**: CDKN2A mutation carriers with metastatic melanoma undergoing BRAF ± MEK or immune checkpoint inhibitors were included in the study and therapy responses assessed. From four publicly available datasets, melanomas with somatic CDKN2A mutation were analyzed for association with tumor mutational load.

Results: Nineteen CDKN2A mutation carriers received BRAF ± MEK inhibitors, thirteen (68%) responded to the therapy, all with a partial response (no complete response) and this was not significantly different from an expected response rate of 64% (13% for complete responses), estimated from the phase III trials involving Dabrafenib, Vemurafenib, Dabrafenib/Trametinib and Encorafenib/Binimetinib 1–3. Nineteen carriers received checkpoint inhibitors, eleven (58%) responded to the therapy which was a significantly higher rate than expected (P = 0.03, binomial test against an expected rate of 37%, estimated from the ipilimumab, pembrolizumab, nivolumab and ipilimumab/nivolumab trials 4–7). Further, six of the nineteen carriers (32%) had complete response which was also a significantly higher rate than expected (P = 0.01, binomial test against an expected rate of 7%). A significantly higher frequency of the CDKN2A mutation carriers receiving immunotherapy had M1c-d disease (79%), brain metastasis (26%) or were previously treated (63%), compared to the patients in the clinical trials. In a separate analysis involving mutation data from 879 tumor samples, the 118 melanomas harboring somatic CDKN2A mutations had significantly higher total numbers of mutations compared to 761 melanomas without CDKN2A mutation (Wilcoxon test, P < 0.001).

**Conclusions**: The response to BRAF ± MEK inhibitors in the germline CDKN2A mutated melanoma patients was not significantly different from an expected response rate. However, the CDKN2A mutated melanoma patients had superior immunotherapy responses and this could be due to increased tumor mutational load in CDKN2A mutated tumors, resulting in more neoantigens and stronger antitumoral immune responses. These findings are primarily reassuring for CDKN2A mutation carriers that have a very high life-time risk to develop melanoma(s), the better than expected response to immunotherapy in the metastatic setting indicates that immunotherapy also could have a significant role in the adjuvant situation among such carriers.

**References**

1. Chapman PB, Hauschild A, Robert C, Haanen JB, Ascierto P, Larkin J, Dummer R, Garbe C, Testori A, Maio M, Hogg D, Lorigan P, et al. Improved survival with vemurafenib in melanoma with BRAF V600E mutation. N Engl J Med 2011;364: 2507–16.
2. Long GV, Stroyakovskiy D, Gogas H, Levchenko E, de Braud F, Larkin J, Garbe C, Jouary T, Hauschild A, Grob JJ, Chiarion Sileni V, Lebbe C, et al. Combined BRAF and MEK inhibition versus BRAF inhibition alone in melanoma. N Engl J Med 2014;371: 1877–88.
3. Dummer R, Ascierto PA, Gogas HJ, Arance A, Mandala M, Liszkay G, Garbe C, Schadendorf D, Krajsova I, Gutzmer R, Chiarion-Sileni V, Dutriaux C, et al. Encorafenib plus binimetinib versus vemurafenib or encorafenib in patients with BRAF-mutant melanoma (COLUMBUS): a multicentre, open-label, randomised phase 3 trial. The Lancet Oncology 2018;19: 603–15.
4. Hodi FS, O’Day SJ, McDermott DF, Weber RW, Sosman JA, Haanen JB, Gonzalez R, Robert C, Schadendorf D, Hassel JC, Akerley W, van den Eertwegh AJ, et al. Improved survival with ipilimumab in patients with metastatic melanoma. N Engl J Med 2010;363: 711–23.
5. Larkin J, Chiarion-Sileni V, Gonzalez R, Grob JJ, Cowey CL, Lao CD, Schadendorf D, Dummer R, Smylie M, Rutkowski P, Ferrucci PF, Hill A, et al. Combined Nivolumab and Ipilimumab or Monotherapy in Untreated Melanoma. N Engl J Med 2015;373: 23–34.
6. Robert C, Long GV, Brady B, Dutriaux C, Maio M, Mortier L, Hassel JC, Rutkowski P, McNeil C, Kalinka-Warzocha E, Savage KJ, Hernberg MM, et al. Nivolumab in previously untreated melanoma without BRAF mutation. N Engl J Med 2015;372: 320–30.
7. Robert C, Schachter J, Long GV, Arance A, Grob JJ, Mortier L, Daud A, Carlino MS, McNeil C, Lotem M, Larkin J, Lorigan P, et al. Pembrolizumab versus Ipilimumab in Advanced Melanoma. N Engl J Med 2015;372: 2521–32.

## Melanoma Bridge 2018

### Emerging strategies session—oral communications

#### O21 Treatment sequencing and survival outcomes in *BRAF* mutation-positive metastatic melanoma patients treated with immunotherapy in routine clinical practice in the United States

##### Natalia Sadetsky^1^, Ching-Yi (James) Chuo^1^, Edward McKenna^1^, Sarah Troutman^1^, Dawn Colburn^1^, Michael B. Atkins^2^

###### ^1^PHC Data Science, Genentech, Inc., South San Francisco, CA, USA; ^2^Department of Medical Oncology, Georgetown University, Washington, DC, USA

*Journal of Translational Medicine* 2019, **17(Supp 1)**:24

**Background:** Advances in the development of selective BRAF inhibitors and newer immunotherapies, such as anti-programmed cell death protein-1 (aPD1), anti-programmed death-ligand 1 (aPD-L1), and cytotoxic T-lymphocyte associated protein 4 (CTLA-4), have led to significant improvements in progression-free and overall survival (OS) for patients with *BRAF*-mutant (mut) metastatic melanoma. However, despite these advances, a significant proportion of the patients progress or do not achieve maximum clinical benefit on first-line therapy.

We aim to describe treatment sequencing and survival outcomes in patients with *BRAF*mut metastatic melanoma treated with immunotherapy in routine clinical practice in the United States.

**Materials and methods:** Patients with *BRAF*mut metastatic melanoma receiving first-line anti-programmed cell death protein-1 (aPD1) therapy (pembrolizumab [PEM], nivolumab [NIVO], or ipilimumab [IPI] + NIVO) between 01/01/2014 and 11/30/2017 were identified (Flatiron Health Data, New York, NY). Patient status was defined as (1) deceased; (2) alive, continuing or completed first-line ≤ 30 days of data cutoff with no evidence of subsequent treatment (second-line); or (3) alive with evidence of second-line.

**Results:** Of 677 *BRAF*mut metastatic melanoma patients treated first line, 192 received aPD-1 therapy: IPI + NIVO, 79 (41%); PEM, 75 (39%); or NIVO, 38 (20%). At data cutoff (01/31/2018), 37 patients (19%) had died. Among 155 patients still alive, 74 patients (39%) were continuing or completed first-line with no evidence of second-line therapy, and 81 patients (42%) had received second-line therapy. Median OS for patients exposed to first-line aPD-1 therapy was not reached (19.8–NA) and varied by lactate dehydrogenase (LDH) level at baseline (LDH high: 13.2 [9.89–20.3] months and LDH low-normal: not reached [22.74 months–NA]). OS with second-line therapy was 13.0 (8.3–NA) months and was also significantly different in LDH normal (19.7 [14.3–NA] months) versus LDH high (7.6 [6.1–NA] months) levels. The most common reasons for discontinuing first-line therapy included disease progression (46 patients [57%]) and treatment toxicity (16 patients [20%]). BRAF-targeted therapy was used as a second-line option in 57 patients (70%).

**Conclusions:** This real-world analysis demonstrated that single or combination aPD-1 therapy was associated with shorter OS in patients with high baseline LDH levels in both first-line or second-line, and that BRAF-targeted therapy was used as a second-line option in a significant proportion of patients following first-line aPD-1 therapy.

#### O22 KEYNOTE-022 part 3: Phase 2 randomized study of 1L dabrafenib and trametinib plus pembrolizumab or placebo for BRAF-mutant advanced melanoma

##### Paolo A. Ascierto^1^, Pier F. Ferrucci^2^, Rosalie Stephens^3^, Michele Del Vecchio^4^, Victoria Atkinson^5^, Henrik Schmidt^6^, Jacob Schachter^7^, Paola Queirolo^8^, Georgina V. Long^9^, Anna Maria Di Giacomo^10^, Inge Svane^11^, Michal Lotem^12^, Gil Bar-Sela^13^, Felix Couture^14^, Bijoyesh P. Mookerjee^15^, Razi Ghori^16^, Nageatte Ibrahim^16^, Blanca Homet Moreno^16^, Antoni Ribas^17^

###### ^1^Melanoma, Cancer Immunotherapy and Development Therapeutics Unit, Istituto Nazionale Tumori Fondazione G. Pascale, Napoli, IT; ^2^Oncology, Istituto Europeo di Oncologia, Milan, IT; ^3^Medical Oncology, Auckland City Hospital, Auckland, NZ; ^4^Medical Oncology, Fondazione IRCCS Istituto Nazionale dei Tumori, Milan, IT; ^5^Medical Oncology, Gallipoli Medical Research Foundation, Greenslopes Private Hospital, Brisbane, QLD, AU; ^6^Oncology, Aarhus University Hospital, Aarhus C, DK; ^7^Oncology, Ella Lemelbaum Institute for Melanoma, Sheba Medical Center at Tel HaShomer, Cancer Center (Oncology Institute), Ramat Gan, IL; ^8^Oncologia Medica 2, IRCCS San Martino-IST, Genova, IT; ^9^Medical Oncology, Melanoma Institute Australia, University of Sydney, North Sydney, NSW, AU; ^10^Medical Oncology, Center for Immuno-Oncology, University Hospital of Siena, Siena, IT; ^11^Oncology, Herlev Hospital, University of Copenhagen, Herlev, DK; ^12^Medical Oncology, Sharett Institute of Oncology, Hadassah Hebrew Medical Center, Jerusalem, IL; ^13^Oncology, Rambam Health Care Campus, Haifa, IL; ^14^Hematology, Centre Hospitalier Universitaire de Québec Research Center, Laval University, Québec, QC, CA; ^15^Oncology Clinical Development, Novartis, East Hanover, US; ^16^Medical Oncology, Merck & Co., Inc., Kenilworth, NJ, US; ^17^Medical Oncology, Ronald Reagan UCLA Medical Center, Los Angeles, CA, US

*Journal of Translational Medicine* 2019, **17(Supp 1)**:25

**Background**: Pembrolizumab + dabrafenib + trametinib had promising antitumor activity and acceptable tolerability in phase 1 of KEYNOTE-022 (NCT02130466).

**Materials and methods:** In the double-blind phase 2 part of KEYNOTE-022, patients with treatment-naive BRAFV600E/K-mutant stage III/IV melanoma were randomly assigned (stratified by ECOG PS [0/1)]; LDH level [> 1.1 vs ≤ 1.1 × ULN]) to pembrolizumab 2 mg/kg Q3 W + dabrafenib 150 mg BID + trametinib 2 mg QD or placebo + dabrafenib + trametinib. Strata with ECOG PS 1 and either LDH level were combined due to small numbers. Primary endpoint was PFS. Significance requirements to reject null hypothesis at 1-sided 0.025 type I error: ~ 74 PFS events for 80% power; observed HR ≤ 0.62. Additional endpoints included ORR, DOR, TTR, and OS. Data cutoff: Feb 15, 2018.

**Results:** Of 60 patients in each arm, most baseline characteristics were balanced (stage IV disease, 98% in pembrolizumab + dabrafenib + trametinib vs 95% in placebo + dabrafenib + trametinib; ECOG PS 0, 80% both; LDH > 1.1 × ULN, 45% vs 43%). Median follow-up for both arms was 9.6 months (range 2.7–23.4). 67% vs 70% received ≥ 12 months of treatment. Median PFS was 16.0 months (95% CI 8.6–21.5) with pembrolizumab + dabrafenib + trametinib vs 10.3 months (95% CI 7.0–15.6) with placebo + dabrafenib + trametinib; HR, 0.66; P = 0.04287; 12-month PFS rates were 59% vs 45%. ORR was 63% vs 72%; CR rates were 18% vs 13%. Median TTR was 2.8 months in each arm; median DOR was 18.7 months (range 1.9+ to 22.1) vs 12.5 (2.1–19.5+). More patients (60%) on pembrolizumab + dabrafenib + trametinib had responses lasting ≥ 18 months vs placebo + dabrafenib + trametinib (28%). OS rates at 12 months were 80% vs 73%. Any grade (G) treatment-related AEs (TRAEs) occurred in 95% vs 93% and G3-5 TRAEs occurred in 58% vs 27% of patients. G3-5 TRAEs occurring in ≥ 5% of patients were pyrexia (10% vs 3%), increased ALT (7% vs 5%), increased AST (8% vs 5%), increased GGT (7% vs 5%), rash (5% vs 2%), and neutropenia (2% vs 5%). 40% vs 20% of patients discontinued any of the 3 study treatments due to TRAEs, and 1 patient died due to a TRAE (pneumonitis) in the pembrolizumab + dabrafenib + trametinib arm. Immune-mediated AEs occurred in 43% vs 13% of patients, most commonly pneumonitis (15% vs 2%), hypothyroidism (8% vs 2%), skin disorders (7% vs 2%), hyperthyroidism (5% vs 0%), and uveitis (5% vs 3%); most resolved with treatment discontinuation/modification.

**Conclusions:** Pembrolizumab + dabrafenib + trametinib vs placebo + dabrafenib + trametinib demonstrated numerically longer PFS and DOR and a higher rate of G3-5 TRAEs in patients with treatment-naive BRAFV600E/K-mutant advanced melanoma.

Clinical trial identification: NCT02130466.

#### O23 Overall survival at 5 years of follow-up from a phase 3 trial comparing ipilimumab 10 mg/kg with 3 mg/kg in patients with advanced melanoma

##### Paolo A. Ascierto^1^, Michele Del Vecchio^2^, Andrzej Mackiewicz^3^, Caroline Robert^4^, Vanna Chiarion-Sileni^5^, Ana Arance^6^, Celeste Lebbé^7^, Inge Marie Svane^8^, Catriona McNeil^9^, Piotr Rutkowski^10^, Carmen Loquai^11^, Laurent Mortier^12^, Omid Hamid^13^, Lars Bastholt^14^, Brigitte Dreno^15^, Dirk Schadendorf^16^, Claus Garbe^17^, Marta Nyakas^18^, Jean-Jacques Grob^19^, Luc Thomas^20^, Gabriella Liszkay^21^, Michael Smylie^22^, Simsek Burcin^23^, Fareeda Hosein^24^, Michele Maio^25^

###### ^1^Melanoma, Cancer Immunotherapy and Innovative Therapy Unit, Istituto Nazionale Tumori Fondazione Pascale, Naples, Italy; ^2^Medical Oncology, National Cancer Institute, Milan, Italy; ^3^Department of Diagnostics and Cancer Immunology, Greater Poland Cancer Centre, Poznan Medical University, Poznan, Poland; ^4^Department of Medicine, Institute Gustave Roussy, Villejuif, France; ^5^Melanoma Cancer Unit, IOV-IRCCS, Padua, Italy; ^6^Hospital Clinic and Institut d´Investigacions Biomèdiques August Pi i Sunyer, Barcelona, Spain; AP-HP Dermatology and CIC Departments, Saint-Louis Hospital, INSERM U976, Université Paris Diderot, Paris, France; ^8^Center for Cancer Immune Therapy, Herlev Hospital, and Department of Oncology, Copenhagen University Hospital, Herlev, Denmark; ^9^Chris O’Brien Lifehouse, Melanoma Institute Australia, and Royal Prince Alfred Hospital, Camperdown, NSW, Australia; ^10^Department of Soft Tissue/Bone Sarcoma and Melanoma, Maria Sklodowska-Curie Institute, Oncology Center, Warsaw, Poland; ^11^Department of Dermatology, University Medical Center, Mainz, Germany; ^12^Clinique de Dermatologie, Unité d’Onco-Dermatologie, INSERM U1189, Centre Hospitalier Régional Universitaire de Lille, Lille, France; ^13^Melanoma Center, The Angeles Clinic and Research Institute, Los Angeles, CA, USA; ^14^Department of Oncology, Odense University Hospital, Odense, Denmark; ^15^Department of Oncodermatology, Nantes University Hospital, Nantes, France; ^16^Department of Dermatology, University Hospital Essen, Essen, and German Cancer Consortium, Heidelberg, Germany; ^17^Department of Dermatology, Eberhard Karls University, Tübingen, Germany; ^18^Department of Oncology, Oslo University Hospital, Oslo, Norway; ^19^Dermatology and Skin Cancers Department, Aix-Marseille University, APHM, Marseille, France; ^20^Department of Dermatology, Centre Hospitalier Lyon-Sud, Pierre-Bénite, France; ^21^National Institute of Oncology, Budapest, Hungary; ^22^Cross Cancer Institute, University of Alberta, Edmonton, AB, Canada; ^23^Global Biometric Sciences, Bristol-Myers Squibb, Princeton, NJ, USA; ^24^Oncology Clinical Development, Bristol-Myers Squibb, Princeton, NJ, USA; ^25^Center for Immuno-Oncology, University Hospital of Siena, Siena, Italy

*Journal of Translational Medicine* 2019, **17(Supp 1)**:26

**Background:** Ipilimumab (IPI) was the first therapy to improve overall survival (OS) in a phase 3 trial of advanced melanoma and has also shown long-term survival (up to 10 years) in approximately 20% of patients [1]. We previously reported the results of a phase 3 trial, which demonstrated significantly longer OS for IPI 10 mg/kg vs IPI 3 mg/kg in patients with advanced melanoma, but with a higher incidence of adverse events (AEs) at 10 mg/kg. Here, we report a 5-year update of OS and safety data from this study.

**Materials and methods:** Eligible patients had unresectable stage III or IV melanoma and had not received prior BRAF or immune checkpoint inhibitors. Patients (N = 727) were randomly assigned 1:1 to IPI 3 mg/kg every 3 weeks (Q3 W) × 4 or IPI 10 mg/kg Q3 W × 4, stratified by M stage, prior treatment, and Eastern Cooperative Oncology Group performance status. Patients who experienced clinical benefit but progressed could be re-induced with IPI at the same dose and schedule. The primary endpoint was OS.

**Results:** At a minimum follow-up of 61 months, an improvement in OS with IPI 10 mg/kg (n = 365) vs IPI 3 mg/kg (n = 362) was sustained, and generally favored IPI at the higher dose in prespecified subgroups (Table 1). Five-year OS rates in the intent-to-treat (ITT) population were 25% and 19%, respectively. Treatment-related grade 3 or 4 AEs were reported in 36% of patients in the IPI 10 mg/kg group and in 20% of patients in the IPI 3 mg/kg group. AEs of any grade led to discontinuation in 34% and 19% of patients, respectively. There were 4 treatment-related deaths for IPI 10 mg/kg and 2 for IPI 3 mg/kg, but no additional treatment-related deaths occurred since the initial analysis.

**Table 1:** Summary of OS data

	Median OS, months (95% CI)		HR (95% CI)
	IPI 10 mg/kg	IPI 3 mg/kg	IPI 10 mg/kg vs IPI 3 mg/kg
ITT population	15.7 (11.6, 17.8)	11.5 (9.9, 13.3)	0.84 (0.71, 0.99)
*BRAF* mutant	33.2 (19.4, 45.2)	19.7 (11.6, 25.3)	0.70 (0.48, 1.02)
*BRAF* wild-type	13.8 (10.2, 17.0)	11.2 (9.2, 13.8)	0.90 (0.73, 1.11)
M0/M1a/M1b	25.9 (22.2, 36.3)	18.7 (15.3, 23.0)	0.77 (0.58, 1.02)
M1c no brain metastases	10.8 (8.2, 16.0)	10.9 (8.3, 13.1)	0.92 (0.71, 1.17)
M1c with brain metastases	7.0 (4.0, 12.8)	5.7 (4.2, 7.0)	0.72 (0.50, 1.05)

**Conclusions:** IPI 10 mg/kg demonstrated a significant improvement in OS vs IPI 3 mg/kg in untreated and previously treated patients with advanced melanoma, which has been sustained after 61 months of follow-up. For both dosing groups, the results suggest the emergence of a plateau in the OS curve, consistent with previous findings observed in IPI studies in advanced melanoma. These results may have implications for the benefit-risk of novel anti-CTLA-4 agents under evaluation.

**Trial registration**: ClinicalTrials.gov, NCT01515189.

**Acknowledgments:** We thank the patients and families who made this trial possible, and the clinical study teams who participated in the trial. This study was funded by Bristol-Myers Squibb. Professional medical writing was provided by Jessica Franciosi, PhD, at StemScientific, funded by Bristol–Myers Squibb.

**Reference**

1. Schadendorf D, Hodi FS, Robert C, et al. Pooled analysis of long-term survival data from phase II and phase III trials of ipilimumab in unresectable or metastatic melanoma. J Clin Oncol. 2015; 33:1889–1894.

#### O24 Phase Ib cohort 1 data of class I HDAC inhibitor 4SC-202 in combination with pembrolizumab in advanced cutaneous melanoma patients refractory or non-responding to prior anti-PD1 therapy

##### Svetlana Hamm, Frank Hermann, René Bartz

###### 4SC AG, Fraunhoferstraße 22, 82152 Planegg-Martinsried; Germany

*Journal of Translational Medicine* 2019, **17(Supp 1)**:27

**Background:** A high proportion of skin cancer patients (e.g. advanced melanoma or merkel cell carcinoma patients) are refractory, do not respond to or relapse on checkpoint inhibition alone, therefore a high unmet medical need remains. One promising approach is to enhance immunogenicity and alter the tumor microenvironment to a more immune-inflamed phenotype by epigenetic intervention. Preclinical experiments suggest various immune-modulatory capabilities of the orally available selective class I HDAC inhibitor domatinostat rendering it as a favorable combination partner for different immunotherapy approaches. Our results provide the rationale and basis for domatinostat as key component for future immune-oncology combination approaches. This concept is currently tested in a Phase Ib multi-center study in advanced melanoma (‘SENSITIZE’; NCT03278665). The SENSITIZE study tests the combination of domatinostat and pembrolizumab in patients not responding to prior immune checkpoint therapy for safety and tolerability, favorable modulation of the tumor microenvironment and clinical efficacy.

**Methods:** Immunomodulatory effects of domatinostat were tested in cell-based assays and various syngenic mouse models. Changes in domatinostat-induced effects on gene expression and direct anti-tumor effects alone or in combination with different immunotherapy approaches were monitored. In SENSITIZE, advanced cutaneous melanoma patients which did not respond to prior checkpoint inhibitor treatment, domatinostat is combined with pembrolizumab to identify the optimal dose for the combination. Tumor assessments are conducted every 12 weeks and sequential tumor biopsies are taken for comprehensive analyses of immune cell infiltration into the tumor. Blood samples are collected in parallel to investigate PK, PD, and changes in gene expression profiles upon treatment.

**Results:** Preclinical characterization demonstrates that domatinostat can modulate the tumor microenvironment leading to an ‘inflamed’ gene expression profile including upregulation of genes essential for antigen presentation and processing. Importantly, combination of domatinostat with different immunotherapy approaches results in synergistic anti-tumor effects. Based on this, the clinical study SENSITIZE has started and all patients for dose cohort 1 (100 mg domatinostat) have been enrolled and the safety review committee (SRC) has recommended the enrolment of patients for dose cohort 2 [200 mg].

**Conclusions:** Based on preclinical experiments suggesting that domatinostat can favorably modulate the tumor microenvironment making it more susceptible for combination for immunotherapy approaches in various indications including advanced melanoma. Our goal is to translate these findings to current SENSITIZE study in patients with advanced melanoma. Domatinostat in combination with pembrolizumab has been shown safe and well tolerated in the lowest dosing cohort (100 mg) and patients are now recruited into dose cohort 2 (200 mg). Biomarker and efficacy data will be taken into consideration to define optimal dosing of domatinostat in combination with anti-PD-1 antibodies.

## Melanoma Bridge 2018

### Posters

#### P4 RNAseq reveals common innate and adaptive resistance mechanisms to BRAF inhibitors

##### Phil F Cheng^1^, Sandra N Freiberger^2^, Anja Irmisch^1^, Reinhard Dummer^1^, Mitchell P Levesque^1^

###### ^1^Dermatology Clinic, University Hospital Zurich, Zurich, Switzerland; ^2^Department of Pathology, University Hospital Zurich, Zurich, Switzerland

*Journal of Translational Medicine* 2019, **17(Supp 1)**:29

BRAF inhibitors have been a great success for patients with a BRAF V600 mutation, however, only about 50% of patients respond to single BRAF inhibitor therapy and the majority of these patients eventually relapse. We have established 53 melanoma cell cultures from biopsies naïve to BRAF inhibitor and progressive on BRAF inhibitor. We in vitro tested each cell line for resistance to BRAF inhibitor and found 27 to be resistant. Surprisingly, a few melanoma cultures from patients who have never been exposed to BRAF inhibitors had innate resistance, while the majority of cell cultures from progressive patients were resistant to in vitro BRAF inhibition. To elucidate the possible resistance mechanisms, we performed RNAseq on all 53 cultures and found differentially expressed genes in common with the innate and adaptive resistance melanoma cell cultures. The phenotype switching signature was one of the resistant mechanisms in common. Although we find some common signatures, there are other resistant cell cultures that have a unique gene signature, which suggests heterogeneity among resistance mechanisms.

#### P5 Long-term survival in metastatic cutaneous melanoma patients treated with NGcGM3/VSSP ganglioside vaccine. Case reports

##### M. Osorio^1^, E. Rodriguez^1^, L. Anasagasti^1^, A. Carr^2^

###### ^1^Clinical Trials Unit. National Institute of Oncology and Radiobiology. Havana, Cuba; ^2^Immunotumoral Direction. Center of Molecular Immunology. Havana, Cuba

*Journal of Translational Medicine* 2019, **17(Supp 1)**:30

For many reasons, advanced melanoma treatment continues to be a challenge up to day in spite of valuable results of new therapy advances, notwithstanding its unquestionable limitations of toxicity and high prices. Different approaches of active specific immunotherapy have been explored many years ago without success. Ganglioside vaccines are one them, with controversial results in the past. NGcGM3/VSSP cancer vaccine was developed at the Center of Molecular Immunology, Havana; Cuba [1]. NeuGcGM3 ganglioside was incorporated in the outer membrane protein complex of *Neisseria meningitidis bacteria* to form very small size proteoliposomes (VSSP). Immunogenicity and low toxicity were evidenced in Preclinical and Clinical studies. NGcGM3 specific antibodies (IgM, IgG and IgA class) [2] and cellular (NK, CD8+ and CD4+ T cells) [3] responses were observed. Recently, inhibition mechanism of Src/FAK/Stat3 pathway was demonstrated [4].

**Case reports**: Clinical evidences of immunogenicity and antitumor response were associated with extended survival times in two patients treated subcutaneously with NGcGM3/VSSP cancer vaccine in a Phase Ib/IIa clinical trial [5]. Vaccine formulation was administered by five doses each 14 days (induction phase) and each 28 days (maintained phase) during 1 year as protocol design. Immunizations continued monthly for four additional years; after that, patients were vaccinated each 3 months. Previously, positive NGcGM3 ganglioside expression was tested in tumor biopsies with specific 14F7 monoclonal antibody (murine IgG1 against NGcGM3) [6]. One patient with lung metastases of cutaneous melanoma that refuses surgery, achieved disease stabilization with excellent quality of life for more than 13 years; another patient with intestinal metastases of amelanotic melanoma, in progressive disease after surgery, obtained complete response sustained for more than 10 years. Transient and reversible local symptoms were observed in both patients. Reversible hypersensibility reaction grade II was observed in the second patient. At the same time, humoral and cellular responses was measured, as signal of certain level of restoration of the patient’s immunocompetence.

**Conclusions**: NGcGM3/VSSP is an attractive target for active specific immunotherapy in melanoma patients, the rate of overall survival observed resembles those with Il-2 therapy. This antitumor responses of long duration with good performance status, constitutes an unusual clinical observation in the frame of a Phase Ib/IIa clinical trial developed. A new scenery for this cancer vaccine in metastatic melanoma patients might be in combination with anti-checkpoint therapy.

**References**

1. Estevez F, Carr A, Solorzano L, Valiente O, Mesa C, Barroso O, Sierra GV, Fernandez LE. Enhancement of the immune response to poorly immunogenic gangliosides after incorporation into very small size proteoliposomes (VSSP). Vaccine. 2000; 18:190–197.
2. Carr A, Rodríguez E, Arango MC, Camacho R, Osorio M, Gabri M, Carrillo G, Valdés Z, Bebelagua Y, Pérez R, and Fernández LE. Immunotherapy of Advanced Breast Cancer with a Heterophilic Ganglioside (NeuGcGM3) Cancer Vaccine. J Clin Oncol. 2003; 21:1015–1021.
3. Labrada M, Clavell M, Bebelagua Y, de León J, Alonso DF, Gabri MR, Veloso RC, Vérez V and Fernández LE. Direct validation of NGcGM3 ganglioside as a new target for cancer immunotherapy. Expert Opin. Biol. Ther. 2010; 10:153–162.
4. González Palomo A, López Medinilla A, Segatori V, Barroso MC, Blanco R, Gabri MR, Carr Pérez A and León Monzón K.Synergistic potentiation of the anti-metastatic effect of anti EGFR mAb by its combination with immunotherapies targeting the ganglioside NGcGM3. Oncotarget. 2018; 9: 24069–24080.
5. Osorio M, Gracia E, Reigosa E, et al. Hernandez J, de la Torre A, Saurez G, Perez K, Viada C, Cepeda M, Carr A, Ávila Y, Rodríguez M, Fernandez LE. Effect of vaccination with N-glycolyl GM3/VSSP vaccine by subcutaneous injection in patients with advanced cutaneous melanoma. Cancer Management and Research. 2012; 4: 341–345.
6. Carr A, Mullet A, Mazorra Z, Vázquez AM, Alfonso M, Mesa C, Rengifo E, Pérez R, and Fernández LE. A Mouse IgG1 Monoclonal Antibody Specific for N-Glycolyl GM3 Ganglioside Recognized Breast and Melanoma Tumors. Hybridoma 2000; 19: 241–247.

#### P6 Patterns of response for combination of PV-10 oncolytic immunotherapy and checkpoint inhibition

##### Sanjiv S. Agarwala^1^, Merrick I. Ross ^2^, Jonathan Zager ^3^, Keisuki Shirai ^4^, Richard Essner ^5^, Bernard M. Smithers ^6^, Victoria Atkinson ^6^, David Sarson ^7^, Eric A. Wachter ^8^

###### ^1^St Luke’s University Hospital and Health Network, Easton, PA USA; ^2^MD Anderson Cancer Center, Houston, TX USA; ^3^Moffitt Cancer Center, Tampa, FL USA; ^4^Dartmouth-Hitchcock Medical Center, Lebanon, NH USA; ^5^Cedars-Sinai Medical Center, Los Angeles, CA USA; ^6^Princess Alexandra Hospital, Brisbane, QLD AUS; ^7^Provectus Biopharmaceuticals Australia Pty Ltd, Sydney, NSW AUS; ^8^Provectus Biopharmaceuticals, Inc., Knoxville, TN USA

*Journal of Translational Medicine* 2019, **17(Supp 1)**:31

**Background:** PV-10 (rose bengal disodium) is the first small molecule oncolytic immunotherapy in development for solid tumors. Intralesional injection can yield immunogenic cell death and tumor-specific reactivity in circulating T-cells [1–4]. PV-10 is currently the subject of a Phase 1b/2 study in combination with systemic immune checkpoint inhibition (CI) for patients with advanced melanoma.

Materials and methods: Study PV-10-MM-1201 (NCT02557321) is assessing PV-10 in combination with pembrolizumab. Patients must have at least 1 injectable lesion, at least 1 measurable target lesion (TL), and be candidates for pembrolizumab. In the Phase 1b portion of the study, patients receive combination treatment during the induction phase (q3w for 5 cycles) and then pembrolizumab alone in the maintenance phase (total duration of up to 24 months); the primary endpoint is safety and tolerability with objective response rate (ORR) and progression-free survival as key secondary endpoints (assessed via RECIST 1.1 after 15 weeks then q12w).

**Results:** An initial Phase 1b cohort of predominantly CI-naïve subjects reached full accrual in April 2018 (20 Stage IV and 3 Stage IIIC/IIID patients). All Treatment-Emergent Adverse Events (TEAEs) were consistent with established patterns for both drugs, with no significant overlap of AEs or unexpected toxicities. All disease stages exhibited response after minimal PV-10 intervention (median 4 cycles of PV-10, range 1–5; median 5 injections of PV-10 per patient, range 1–82), with 9% complete response (CR) and 65% ORR (overall per RECIST) as of a 1 Nov 2018 data cutoff. Response of injected target lesions (77% CR and 80% ORR across all disease stages) was higher than historical data for single-agent PV-10 (46% CR and 53% ORR across all disease stages). Although data on the combination for treatment of Stage III (M0) disease is currently limited, response rate of injected target lesions was also higher for M0 disease than observed in single-agent use: 67% CR (4 of 6 lesions) vs 54% CR (214 of 395 lesions).

**Conclusion:** Robust response was observed across all disease stages. A first expansion cohort is accruing CI-refractory patients to further characterize response in this emergent population. Systemic therapy with CI is now recommended in the USA for Stage III patients with satellite or in-transit disease [5], but KEYNOTE-001 demonstrated lower overall response in M0 vs M1 patients [6] and for subcutaneous vs visceral lesions [7]. To address this population, a second expansion cohort directed to patients with satellite or in-transit disease will be opened in early-2019.

**References**

1. Wachter E, Dees C, Harkins J, Fisher W, Scott T. Functional imaging of photosensitizers using multiphoton microscopy. Proceedings of SPIE, Multiphoton Microscopy in the Biomedical Sciences II, Periasamy, A. and So, P.T.C. (eds), Bellingham, Washington: 2002; 4620:143–147.
2. Liu H, Innamarato PP, Kodumudi K, Weber A et al. Intralesional rose bengal in melanoma elicits tumor immunity via activation of dendritic cells by the release of high mobility group box 1. Oncotarget 2016; 7:37893–37905.
3. Qin J, Kunda N, Qiao G, Calata JF et al. Colon cancer cell treatment with rose bengal generates a protective immune response via immunogenic cell death. Cell Death and Disease 2017; 8:e2584, 10.1038/cddis.2016.473.
4. Liu H, Weber A, Morse J, Kodumudi K et al. T cell mediated immunity after combination therapy with intralesional PV-10 and blockade of the PD-1/PD-L1 pathway in a murine melanoma model. PLoS One 2018; 13:e0196033. 10.1371/journal.pone.0196033.
5. NCCN Clinical Practice Guidelines in Oncology, Cutaneous Melanoma. National Comprehensive Cancer Network, Ver 1.2019, Nov 2018.
6. Ribas A, Hodi FS, Kefford R, Hamid O et al. Efficacy and safety of the anti-PD-1 monoclonal antibody MK-3475 in 411 patients (pts) with melanoma (MEL). J Clin Oncol. 2014; 32:5s (suppl; abstr LBA9000^).
7. Lee JHJ, Lyle M, Menzies AM, Chan MMK et al. Metastasis-specific patterns of response and progression with anti-PD-1 treatment in metastatic melanoma. Pigment Cell Melanoma Res. 2018; 31:404–410.

## Melanoma Bridge 2018

### Speaker keynotes

#### K2 The new era of adjuvant therapies for melanoma: changes in management

##### Alexander M. M. Eggermont

###### Gustave Roussy CCC, Villejuif, France

*Journal of Translational Medicine* 2019, **17(Supp 1)**:28

With the effective recently approved drugs in advanced melanoma (1) we have witnessed within a time span of only 4 years (2015–2018) the results of 4 randomized controlled trials (RCT) demonstrating a significant impact on recurrence-free survival (RFS) for adjuvant therapy with ipilimumab (2) resulting also in a similar overall survival benefit (3); for nivolumab (4); for dabrafenib plus trametinib (5) and for pembrolizumab (6).

**Consistency across trials:** Across the trials EORTC18071/CA-029 trial of ipilimumab versus placebo in stage IIIA(> 1 mm)/B/C, the Checkmate-238 trial nivolumab versus ipilimumab in stage IIIB/C-IV, the Combi-AD (dabrafenib plus trametinib versus plac*ebo) trial in stage IIIA(*> *1* *mm)/B/C, and the EORTC 1325/Keynote*-*054 (pembrolizumab versus placebo) trial in stage IIIA(*> *1* *mm)/B/C,* a striking consistency of outcomes is observed. Ipilimumab has a modest but significant impact on RFS, with a HR of 0.75, and RFS rates at 12 and 18 months that are 9% and 8% better for ipilimumab. At 5 years RFS rates were 11% better for RFS as well as for OS. Nivolumab, pembrolizumab and the combination of dabrafenib plus trametinib have all a superior impact. Nivolumab is superior to ipilimumab with a HR of 0.65 and RFS rates at 12 and 18 months 10% and 11% better than for ipilimumab. The curves are a bit lower than in the other 3 trials because the trial population stage IIIB/C-IV has poorer prognosis than the population studied in the 3 other trials (stage III! > 1 mm/B/C). Interestingly, in the EORTC 18071 trial of ipilimumab, the 50% recurrence-free survival rate at 18 months in the placebo group was almost identical to that observed in the placebo group in the EORTC 1325 trial of pembrolizumab and very similar to the placebo arm of the Combi A-D trial. The ipilimumab benefit over placebo was not as large (hazard ratio 0.76; 18-month RFS rate difference of only 8%; 57% for ipilimumab vs 49% for placebo), whereas in the pembrolizumab trial the HR was 0.57, with a RFS rate difference at 18 months of 18%. This indicates that also pembrolizumab is clearly more effective than ipilimumab, which is in line with the superiority of nivolumab over ipilimumab in the CheckMate 238 trial (18-month RFS rate difference of 14% over ipilimumab. Further credence to the great consistency of the data of these trials is that in the overlapping stage IIIB/C patient populations, the 18-month RFS rates were virtually identical: 72.2% and 72.3% for pembrolizumab and for nivolumab respectively. The Combi-AD trial, comparing the combination of dabrafenib and trametinib with placebo in patients with stage IIIA(> 1 mm)/B/C melanoma with a BRAF-V600E/K mutation demonstrated also a highly significant benefit with a HR of 0.47 and 12 months and 18 months RFS-rate differences of 32% and 31% respectively.

**Treatment-related adverse events (AEs):** Ipilimumab was clearly associated with most treatment related AEs (94%. Immune related Adverse Events (irAEs) occurred in 90% of patients, grade 3–4 irAEs in 43.5% (most important: diarrhea/colitis 15%, Hepatitis 12%, hypophysitis 4.8%) with 5 patients who died of colitis, myocarditis, guillain–barre syndrome. In sharp contrast both the nivolumab and the pembrolizumab trial demonstrated very similar and favourable side-effect profiles with treatment-related AEs in about 14% of patients and irAEs in only 7% of patients. In the pembrolizumab trial there was one grade 5 case (myositis), in the nivolumab trial zero. Relatively frequent was grade 1–2 thyroid-endocrinopathy (20%) that was easy to treat. IrAEs grade 3–4 events were rare in both trials: colitis (2%), hepatitis (1.4%), diabetes (1%), pneumonitis (0.8%) hypophysitis (0.6%) nephritis (0.4%). Dabrafenib plus trametinib in the was associated with more AEs than the anti-PD1 trials, but less than the ipilimumab trial. The dabrafenib plus trametenib combination was associated with pyrexia grade 1–2 in 97% with chills in 37%, and grade 3–4 pyrexia in 5%. Grade 3–4 events occurred in 41% of the patients, a.o. hypertension (6%), fatigue (4%), hepatitis (4%). Drug related AEs lead to drug discontinuation in 50% of patients with ipilimumab, in 26% with dabrafenib-trametinib and in only 14% with nivolumab or pembrolizumab.

**The new adjuvant landscape with simplified staging requirements:** A new adjuvant therapy landscape for high-risk melanoma has emerged with pembrolizumab and nivolumab as effective agents along with the combination of dabrafenib and trametinib as an additional option for *BRAF*-mutant melanoma. The results put an end to adjuvant therapy with ipilimumab as well as to the use of interferons, whose use can be limited to patients with ulcerated melanomas in countries without access to the new therapies (7). Although completion lymph-node dissection (CLND) has been a mandatory component in all adjuvant phase 3 trials to date, it is no longer considered mandatory based on the results of the MSLT-II and DeCOG CLND trials.(8,9), and can be further simplified by combining SN tumor load information and the (non) ulcerated status of the primary melanoma (10).

**Next step: neoadjuvant strategies:** Further clinical development may involve neoadjuvant use of pembrolizumab or nivolumab alone or in combination with ipilimumab, or a BRAF/MEK inhibitor combination, especially attractive in palpable nodal stage III disease (12,13).

**References**

1. Ugurel S, Röhmel J, Ascierto PA, et al. Survival of patients with advanced metastatic melanoma: the impact of novel therapies-update 2017. Eur J Cancer. 2017;83:247–257.
2. Eggermont AM, Chiarion-Sileni V, Grob JJ, et al. Adjuvant ipilimumab versus placebo after complete resection of high-risk stage III melanoma (EORTC 18071): a randomised, double-blind, phase 3 trial. Lancet Oncol 2015;16:522–30.
3. Eggermont AM, Chiarion-Sileni V, Grob JJ, et al. Prolonged survival with Ipilimumab as adjuvant in stage III melanoma. New Engl J Med 2016;375:1845–1855.
4. Weber J, Mandala M, Del Vecchio M, et al. Adjuvant Nivolumab versus Ipilimumab in Resected Stage III or IV Melanoma. N Engl J Med. 2017;377:1824–1835.
5. Long GV, Hauschild A, Santinami M, et al. Adjuvant Dabrafenib plus Trametinib in Stage III BRAF-Mutated Melanoma. N Engl J Med. c.
6. Eggermont AMM, Blank CU, Mandala M, et al. Adjuvant Pembrolizumab versus Placebo in Resected Stage III Melanoma. N Engl J Med. 2018 May 10;378(19):1789–1801.
7. Ives NJ, Suciu S, Eggermont AMM, et al., on behalf of the International Melanoma Meta-Analysis Collaborative Group (IMMCG). Adjuvant interferon-α for the treatment of high-risk melanoma: An individual patient data meta-analysis. Eur J Cancer. 2017;82:171–183.
8. Leiter U, Stadler R, Mauch C, et al. Complete lymph node dissection versus no dissection in patients with sentinel lymph node biopsy positive melanoma (DeCOG-SLT): a multicentre, randomised, phase 3 trial. Lancet Oncol. 2016;17(6):757–67.
9. Faries MB, Thompson JF, Cochran AJ, et al. Completion Dissection or Observation for Sentinel-Node Metastasis in Melanoma. N Engl J Med. 2017;376(23):2211–2222.
10. Verver D, van Klaveren D, van Akkooi ACJ, et al. Risk stratification of sentinel node-positive melanoma patients defines surgical management and adjuvant therapy treatment considerations. Eur J Cancer. 2018;96:25–33.

